# The Integration of Fire Ecology and Freshwater Ecosystems in North America: Knowledge Gaps and Research Needs

**DOI:** 10.1111/gcb.70945

**Published:** 2026-06-16

**Authors:** Morgan L. Piczak, Ava J. A. Sergio, Caliyena R. Brown, Waverley S. Birch, Rebecca L. Flitcroft, Meg A. Krawchuk, Colin P. R. McCarter, Jonathan W. Moore, Brendan P. Murphy, Brooke E. Penaluna, Prabha A. Rupasinghe, Victoria Steblaj, Sophie L. Wilkinson, Chantel E. Markle

**Affiliations:** ^1^ Department of Forest and Conservation Sciences, Faculty of Forestry and Environmental Stewardship University of British Columbia Vancouver British Columbia Canada; ^2^ Department of Biology, Life Science Centre Dalhousie University Halifax Nova Scotia Canada; ^3^ Department of Geography and Environmental Management University of Waterloo Waterloo Ontario Canada; ^4^ USDA Forest Service Pacific Northwest Research Station Corvallis Oregon USA; ^5^ Department of Forest Ecosystems and Society, College of Forestry Oregon State University Corvallis Oregon USA; ^6^ Department of Biology, Chemistry, and Geography Nipissing University North Bay Ontario Canada; ^7^ Earth to Ocean Research Group Simon Fraser University Burnaby British Columbia Canada; ^8^ School of Environmental Science Simon Fraser University Burnaby British Columbia Canada; ^9^ Independent Researcher Calgary Alberta Canada; ^10^ School of Resource and Environmental Management Simon Fraser University Burnaby British Columbia Canada; ^11^ School of Environment, Resources and Sustainability University of Waterloo Waterloo Ontario Canada

**Keywords:** biodiversity, fire management, freshwater, wildland fire

## Abstract

Wildfires, unplanned fires that start and spread under the influence of weather and fuel composition, are increasing in frequency, duration, and severity across North America. While wildfires are one component of wildland fire, a broader natural process in many ecosystems, their changing behavior poses growing ecological and management challenges. Although wildfire is widely recognized as an important disturbance, empirical research quantifying its impacts on freshwater ecosystems remains limited and fragmented. As a result, our understanding of how wildfire affects imperiled freshwater systems remains underdeveloped. The objective of this review was to synthesize the existing empirical literature and identify key knowledge gaps to guide future research on wildfire–freshwater interactions across both biotic and abiotic components. At the biological scale, uncertainties remain around species‐ and community‐level responses (e.g., direct and indirect effects on biota). Freshwater habitat dimensions should also be the focus of further research, including alterations to physical structure (e.g., vegetation) and physicochemical conditions (e.g., temperature). From a management perspective, critical questions relate to fire‐informed restoration (e.g., specialized techniques, role of managed burns) and strategies for enhancing ecosystem resilience (e.g., identifying relevant indicators, links to watershed health). Understanding how land‐use change (e.g., forestry) and climate change interact to influence wildfire regimes and generate cumulative effects across landscapes is also essential in this new wildfire reality. To advance research and practice, we recommend comprehensive monitoring, standardization of methods and indicators, recognition of the role of cultural and prescribed burns, meaningful knowledge co‐production, and bridging the gap between scientific knowledge and management action.

## Graphical abstract

Illustration of key knowledge gaps and pathways linking fire ecology and freshwater ecosystems. Climate change provides the overarching context, interacting with cumulative and cross‐scale effects to shape local freshwater conditions. Within this context, fire characteristics influence three primary pathways within freshwater ecosystems: species and community responses, habitat structure, and physicochemical conditions. Advancing understanding across these pathways supports fire‐informed restoration and strategies for building freshwater ecosystem resilience.

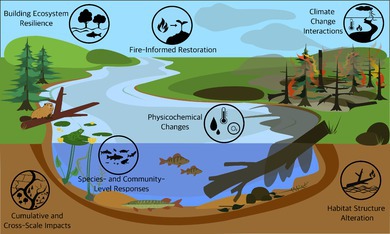

## 1 Introduction

Wildfires are a natural ecological process that many ecosystems have adapted to over evolutionary time (Keeley and Pausas 2022). In some fire‐adapted landscapes, burns occurring at frequencies consistent with historical fire regimes (see Table 1 for glossary) maintain ecosystem function and promote biodiversity (He et al. 2019). However, the most serious concerns today stem not from fire itself, but from shifting fire regimes (Jones et al. 2022), particularly the increasing intensity, frequency, spatial extent, and synchronicity of high‐severity fires (see Table 1 for glossary), as well as shifts in seasonal timing that depart from historical fire regimes in many ecosystems. Wildfires are one type of wildland fire which is a broad category that includes both planned and unplanned fires that burn natural fuels. Wildland fires generally encompass wildfire, cultural burning, and prescribed fire (see glossary in Table 1), but in this synthesis, we focus specifically on wildfires due to their growing ecological impacts and global relevance.

**TABLE 1:** Key terminology used throughout this perspective.

Term	Definition	References
Burn severity	A qualitative assessment of the heat pulse directed toward the ground during a fire. Burn severity relates to soil heating, large fuel and duff consumption, consumption of the litter and organic layer beneath trees and isolated shrubs, and mortality of buried plant parts	US Forest Service Burned Area Emergency Response (BAER) Glossary
Cultural fire	Deep‐rooted Indigenous‐led practice with cultural, spiritual, and ecological goals, often focused on land stewardship and resource sustainability	Parks Canada (2025)
Experimental fires	Burns intentionally ignited to study the behavior and ecological effects of natural wildfires under more controlled conditions	Alexander and Quintilio (1990)
Fire Effects	The physical, biological, and ecological impacts of fire on the environment. Two types are often discussed: first‐order fire effects (direct effects of the combustion process on the environment) and second‐order fire effects (effects that occur after some time and are often caused by the interaction of fire‐caused stress with other factors).	Parsons et al. (2010)
Fire Intensity	The amount of energy or heat released per unit time or area during the consumption of organic matter	Keeley (2009)
	Degree to which a site has been altered or disrupte	
First‐order fire effects	First‐order fire effects occur during a fire or immediately after (e.g., within minutes, hours, days); referred to as direct effects of fire	Higuera (2019)
Fuel break	A barrier or a change in fuel type or condition (to one that is less flammable than that surrounding it), or a strip of land that has been modified or cleared to prevent fire spread. In the event of fire, it may serve as a control line from which to carry out suppression operations	https://www2.gov.bc.ca/gov/content/safety/wildfire‐status/about‐bcws/glossary
Historical natural fire regime	Historical fire conditions under which vegetation communities presumably evolved and were maintained. Reflect typical fire frequencies and effects that evolved without fire exclusion	Hardy (2005)
Ladder fuel	Fuels that provide vertical continuity between the surface fuels and crown fuels in a forest stand, thus contributing to the ease of torching and crowning (e.g., tall shrubs, small‐sized trees, bark flakes, tree lichens)	Government of BC, Canada https://www2.gov.bc.ca/gov/content/safety/wildfire‐status/about‐bcws/glossary
Prescribed Fire	Agency led practice focused on fuel management, hazard reduction, and public safety that uses ignition methods such as gasoline or drop torches	Parks Canada (2025)
Resilience	The ability to absorb disturbances and reorganize under change to maintain similar functioning and structure	Scheffer (2009)
Second‐order fire effects	Second‐order fire effects occur over the weeks, months, and years after fire; referred to as indirect effects	Higuera (2019)
Soil Burn Severity	The effect of a fire on ground surface characteristics, including char depth, organic matter loss, altered color and structure, and reduced infiltration	Parsons et al. (2010)
Surface fuel	All combustible materials lying above the duff layer between the ground and ladder fuels that are responsible for propagating surface fires (e.g., litter, herbaceous vegetation, low and medium shrubs, tree seedlings, stumps, downed‐dead roundwood)	https://www2.gov.bc.ca/gov/content/safety/wildfire‐status/about‐bcws/glossary
Vegetation Burn Severity	The effect of a fire on vegetative ecosystem properties, often defined by the degree of scorch, consumption, and mortality of vegetation and the projected or ultimate vegetative recovery	Parsons et al. (2010)
Wildfire	A fire in wildlands that is unplanned and uncontrolled	National Park Service (2025)
Wildland fire	Both planned and unplanned fires that consume natural fuels	Parks Canada (2025)

Wildfires are driven in part by the accelerating effects of climate change, such as rising air temperatures, fuel aridity, shifts in precipitation patterns, and increasing droughts (Rogers et al. 2020; Kirchmeier‐Young et al. 2020). The characteristics of historical fire regimes are shifting, with wildfire seasons starting earlier, lasting longer, and producing larger and more severe burns than in the past (Krawchuk et al. 2009; Hanes et al. 2019; Bowman et al. 2020; McFarland et al. 2025). These shifts are not solely attributable to climate change in that land use changes (e.g., agriculture, logging; Lindenmayer et al. 2022), anthropogenic stressors (e.g., biological invasions; Romualdi et al. 2023), systematic erasure of Indigenous fire stewardship (Bowman et al. 2011; Hoffman et al. 2021), continued wildfire suppression, and associated fuel accumulation over decades of wildfire exclusion have all contributed to the increasing prevalence of uncharacteristically large and severe wildfires (Baron et al. 2022; Daniels et al. 2024; Kreider et al. 2024). These altered fire regimes can disrupt ecological processes and lead to significant declines in biodiversity, particularly when species or habitats are ill‐adapted to such novel disturbance patterns (Kelly et al. 2020). While the impacts of wildfires on terrestrial ecosystems have received substantial attention (e.g., Wan et al. 2001; Geary et al. 2020; McLauchlan et al. 2020), the effects of wildfire on freshwater ecosystems remain poorly understood (Gomez Isaza et al. 2022; Erdozain et al. 2024; Bixby et al. 2015).

Over the last few years, research has highlighted knowledge gaps that hinder our understanding of the effects of wildfire on terrestrial (e.g., Sanderfoot et al. 2021), marine (e.g., Riera and Pausas 2024), estuarine (e.g., Laicher et al. 2026), and freshwater ecosystems (e.g., Gomez Isaza et al. 2022; Paul et al. 2022; Murphy et al. 2023). However, biodiversity loss in freshwater ecosystems exceeds losses relative to marine or terrestrial systems (Tickner et al. 2020) resulting in many freshwater ecosystems facing crises of both biodiversity loss and climate change. The main threats facing freshwater biodiversity include overexploitation, water pollution, flow modification, invasive species, habitat loss, habitat alteration (Dudgeon et al. 2006), and shifts in hydrological connectivity (Tickner et al. 2020). It is essential to mitigate these stressors and their cumulative effects facing freshwater ecosystems, as they not only support disproportionately high biodiversity (Strayer and Dudgeon 2010), but also provide crucial ecosystem services that benefit human societies (Lynch et al. 2016). While wildfires are a form of habitat alteration, their impacts differ from anthropogenic activities. Wildfire has historically been a natural process where fire‐induced changes occur rapidly and often abruptly compared with disturbances such as industrial development or biological invasions (Anderson 2014). Wildfire effects on freshwater ecosystems reflect complex landscape, management, and environmental interactions, therefore requiring tailored management approaches (Pacific Salmon Foundation 2024). Some of the known impacts on freshwater from wildfire can include channel reconfiguration, increased sedimentation and large woody debris, nutrient loading, altered hydrology and internal biogeochemical cycling, changes in groundwater and surface water interactions, and the disruption of aquatic food webs in affected streams or watersheds (reviewed in Bixby et al. 2015; Gresswell 1999; Jager et al. 2021; Rey et al. 2023). Despite past research, there is still a limited understanding of wildfire impacts, hindering effective management of these disturbances in freshwater ecosystems (Gomez Isaza et al. 2022; Paul et al. 2022; Murphy et al. 2023). As climate change and land‐use change compound to increase the frequency and severity of wildfires, there is an urgent need to better understand their consequences for freshwater systems and to integrate these insights into conservation and management strategies.

Historically, the fields of wildfire ecology and freshwater ecology have developed as separate disciplines with unique journals, tools, and terminology that have reinforced the continued separation of ideas. Despite growing recognition of wildfire as a major disturbance, empirical research quantifying its impacts on freshwater ecosystems remains limited, fragmented, and uneven across taxa, ecosystems, and study designs. It is therefore imperative not only to integrate ideas from wildfire and freshwater ecology, but also to critically evaluate what we have learned and, importantly, what remains unknown. This perspective proposes a framework to link research and management priorities to support freshwater ecosystem resilience given increases in fire, highlighting both positive and negative outcomes associated with wildfire. Here, we synthesize our current understanding of the impacts of wildfires on North American freshwater ecosystems, defined here as inland aquatic systems including streams, rivers, lakes, ponds, wetlands (i.e., marshes, swamps, fens, bogs, peatlands), as well as floodplains and riparian zones, and the underlying ecological processes that link wildfire and freshwater ecosystems to help guide future efforts. We also identify seven critical knowledge gaps that reflect not only areas of uncertainty, but also fundamental limitations in the scope, scale, and mechanistic understanding of existing research, including gaps in taxonomic and geographic coverage, study design, and integration with fire regime characteristics. Finally, we highlight methodological and interdisciplinary opportunities as well as solutions to address these gaps. Our goal is to first prioritize research to advance our understanding and management of wildfire and freshwater ecosystems and second, to ultimately generate robust knowledge so that decision‐making and management can be rooted in evidence.

## 2 A Primer: How Fires Are Studied in Freshwater Ecosystems

A variety of study approaches, ranging from opportunistic to experimental, have been used to understand the ecological effects of wildfire on freshwater ecosystems. The study of wildfire is inherently opportunistic, due to the unpredictable nature of wildfire timing and extent. Consequently, Control‐Impact and After‐Only are often the most feasible study designs for documenting impacts associated with fires. Control‐Impact study designs make comparisons between a burned site (impact) and an unburned reference site (control; e.g., Markle, Wilkinson, and Waddington 2020); whereas After‐Only studies are completed post hoc, where data are collected only once the wildfire has occurred (e.g., Carvalho et al. 2019; Rust et al. 2019). Both approaches can limit the strength of inferences and conclusions gleaned from these data as they inherently make the assumption that burned and unburned sites are comparable. Although Before‐After‐Control‐Impact (BACI) study designs offer a much stronger framework for detecting fire‐driven ecological change, they are less common in wildfire research because of the fundamental challenge of having pre‐fire data on burned and unburned locations.

Cultural and prescribed burns offer greater control than natural fires, and because researchers know when and where they will occur, these burns can support more robust study designs (Hoffman et al. 2021). Cultural burning is Indigenous‐led and is distinct from prescribed burning in the techniques used to burn and the primary objectives of the burn (Hoffman, Christianson, Dickson‐Hoyle, et al. 2022; see Table 1 for glossary). Varied burning practices have been used for ceremonial purposes and as a resource management approach by Indigenous Peoples for millennia (Hoffman, Christianson, Dickson‐Hoyle, et al. 2022), often to maintain habitat heterogeneity, support culturally important species, and sustain ecosystem function across landscapes that include riparia and wetland environments (Kimmerer and Lake 2001; Bowman et al. 2011; Hoffman et al. 2021). On the other hand, prescribed burning is often agency‐led (e.g., Parks Canada or United States Forestry Service) and used for restoring natural wildfire disturbance regimes, fuel management, and to modify vegetation structure and composition to enhance ecosystem resilience to future wildfire (see Table 1 for glossary), and is therefore regarded as an ecological surrogate for natural fires (McIver et al. 2008). Most prescribed fires tend to be low intensity and severity (Hahn et al. 2019; see Table 1 for glossary), purposefully occurring under different fire weather conditions than wildfires (Benali et al. 2017) that can constrain the ecological and hydrogeomorphic insights that can be drawn from their study. Nonetheless, prescribed burns have been used to study post‐fire effects on the physical environment, biogeochemical variables, and species richness for freshwater taxa ranging from macroinvertebrates to fish and amphibians (Caldwell et al. 2013; Arkle and Pilliod 2010). Additionally, the intentionality of both cultural and prescribed burns allows for the collection of baseline ecosystem data before burning, facilitating robust BACI study designs in a natural setting. Different from both cultural and prescribed burns are “experimental fires” (see Table 1 for glossary); these fires are typically agency‐led (similar to prescribed burns), yet the goals are to replicate or imitate the wildfire behavior and effects of a natural wildfire (i.e., under moderate to high wildfire weather conditions). Experimental fires have been successfully used to conduct BACI study designs in peatlands (e.g., Wilkinson, Moore, Thompson, et al. 2018; Thompson et al. 2020), ponds (Kelly and Harris 2024), and lakes (Gschwentner et al. 2025), although these studies remain relatively rare due to large resource requirements, complex logistical planning, the tendency not to implement managed riparian burns, and constrained fire weather conditions. Even then, these burns are seldom conducted under truly severe fire weather, as safety constraints and operational limits prevent replicating the most extreme, and ecologically consequential, fire scenarios.

Mesocosms and lab‐based studies provide controlled experimental settings for investigating the effects of wildfire on freshwater ecosystems. These approaches have been applied to examine the effects of post‐fire ash on the physiological tolerances of freshwater fauna (e.g., Gonino et al. 2019), and water chemistry (Cramp et al. 2021; Earl and Blinn 2003). Their relatively small scale compared to in situ burning enables the detection of fine‐scale changes in processes such as nutrient, element, and carbon cycling in freshwater (Earl et al. 2025; Wall et al. 2025). While useful for determining the mechanisms impacted by wildfire, processes and impacts derived from mesocosm and lab‐based studies often are not transferable to larger spatial scales since it is difficult to incorporate the host of interconnected processes that govern ecosystem or catchment responses.

Paleolimnology and other paleoclimate methods have also been used to study the effects of both wildfire and prescribed burns in freshwater ecosystems (Pelletier et al. 2020; Paterson et al. 2002; Charette and Prepas 2003; Waters et al. 2023; Enache and Prairie 2000). Paleolimnological methods are effective for determining the historical long‐term effects of fire disturbance on freshwater ecosystems. These methods have been used to evaluate changes in nutrient deposition (Waters et al. 2023), phytoplankton communities (Charette and Prepas 2003; Waters et al. 2023; Paterson et al. 2002), and element fluxes (Pelletier et al. 2020) to better understand the overarching shifts in freshwater biogeochemistry post‐fire (Enache and Prairie 2000). It is important to note that paleolimnological approaches have limitations, as fire‐related signals can be difficult to detect depending on sedimentation rates and core resolution, and it is often challenging to disentangle wildfire effects from concurrent climatic and environmental drivers.

Remote sensing technology provides valuable spatial information about the Earth's surface and its dynamic processes. A variety of remotely‐sensed data types, including optical imagery (multispectral and hyperspectral), thermal infrared, radar, and Light Detection and Ranging (LiDAR), are widely used to study freshwater ecosystems, including lentic and lotic systems and wetlands and wildfires, particularly across broad spatial and temporal scales (Leblon et al. 2012; Guo et al. 2017; Chasmer et al. 2020; Dang et al. 2021). These datasets enable BACI study designs through the assessment of pre‐fire, active fire, and post‐fire conditions. Pre‐fire assessments may include fuel content and moisture levels, vegetation health and structure, biomass, species composition, drought conditions, and wetland water level (Leblon et al. 2012; Guo et al. 2017). In freshwater ecosystems, particularly in lentic systems such as lakes and wetlands, optical and hyperspectral remote sensing is also increasingly used to derive aquatic‐specific metrics such as lake and wetland color, turbidity, suspended sediment concentration, chlorophyll‐*a*, dissolved organic matter, and surface temperature, which provide insights into primary productivity, nutrient dynamics, and water quality conditions (Kutser 2009; Odermatt et al. 2012; Palmer et al. 2015). Thermal imagery, often combined with optical data, is useful for monitoring active wildfire attributes such as flame length and height, fire temperature, rate of spread, and energy output (Lentile et al. 2006). There has also been an increasing effort to monitor fires live through online platforms such as the National Atmospheric and Space Agency's Fire Information for Resource Management System (https://firms.modaps.eosdis.nasa.gov/) and Natural Resource Canada's Canadian Wildland Fire Information System (https://cwfis.cfs.nrcan.gc.ca/home). In addition, the National Oceanic and Atmospheric Administration's (NOAA) Hazard Mapping System (HMS) Fire and Smoke Plume product provides near‐real‐time information on wildfire locations and smoke plume extent using multisensor satellite observations. Smoke plume data are increasingly recognized as important for freshwater research, particularly in lakes and wetland systems where reduced light availability, altered thermal regimes, and atmospheric deposition of nutrients and contaminants can directly influence water quality and biological processes (Scordo et al. 2021; Farruggia et al. 2024). Lastly, post‐fire attributes commonly studied include burn severity and extent, vegetation and soil changes, shifts in water levels and quality, patterns and rates of ecosystem recovery (Leblon et al. 2012; Li et al. 2020; Stankova 2023), and erosion and hazard mapping and characterization (Orem and Pelletier 2015; Rengers et al. 2024; Ridgway et al. 2024; Hancock and Wlodarczyk 2025). Post‐fire remote sensing of freshwater systems can further capture changes in lentic systems such as water color, turbidity, algal biomass, and light penetration resulting from ash deposition, sediment influx, and smoke‐related atmospheric attenuation, while in lotic systems remote sensing is more commonly used to assess sediment transport, channel changes, and connectivity, reflecting differences in how these systems respond to wildfire. Given its broad applicability and capacity to assess large areas over time, including both pre‐fire conditions and post‐fire recovery, remote sensing is an increasingly powerful tool in wildfire research in both terrestrial and freshwater ecosystems.

Predicting wildfire occurrence and behavior is a crucial component of wildfire management. Modeling approaches are increasingly used in freshwater ecosystems, particularly in wetlands and forested catchments where wildfires can alter hydrological regimes, groundwater recharge, and surface water dynamics, thereby influencing habitat conditions and water quality. Wildfire is a complex phenomenon influenced by numerous interacting factors, including weather conditions, fuel type and load, fuel moisture content, topography, vegetation characteristics, and the means of ignition (Bakhshaii and Johnson 2019). Wildfire modeling integrates these variables through mathematical formulas and algorithms to simulate wildfire dynamics (Morvan 2019). Models are generally classified into two main categories: physical and empirical. Physical models consider combustion chemistry, heat transfer, and fluid dynamics based on fundamental physics and chemistry (Sullivan 2009), while empirical models rely on statistical relationships derived from observed or experimental wildfire data (Sullivan 2009). Contemporary wildfire models integrate both approaches and also account for fire–atmosphere interactions, improving accuracy (Bakhshaii and Johnson 2019). Advances in numerical methods, remote sensing, meteorological forecasting, computer sciences, and computational power have significantly enhanced the performance and predictive capacity of wildfire models (Bakhshaii and Johnson 2019; Jain et al. 2020; Singh et al. 2024; Klimas et al. 2025). These developments are enabling more effective wildfire management strategies by providing spatially and temporally explicit wildfire behavior forecasts and risk assessments. Continued improvements in modeling are especially critical in the context of climate change, which is altering fire regimes and increasing wildfire risks across multiple freshwater ecosystems such as wetlands and shallow lakes (Rosa et al. 2023).

## 3 Knowledge Gaps

**FIGURE 1:**
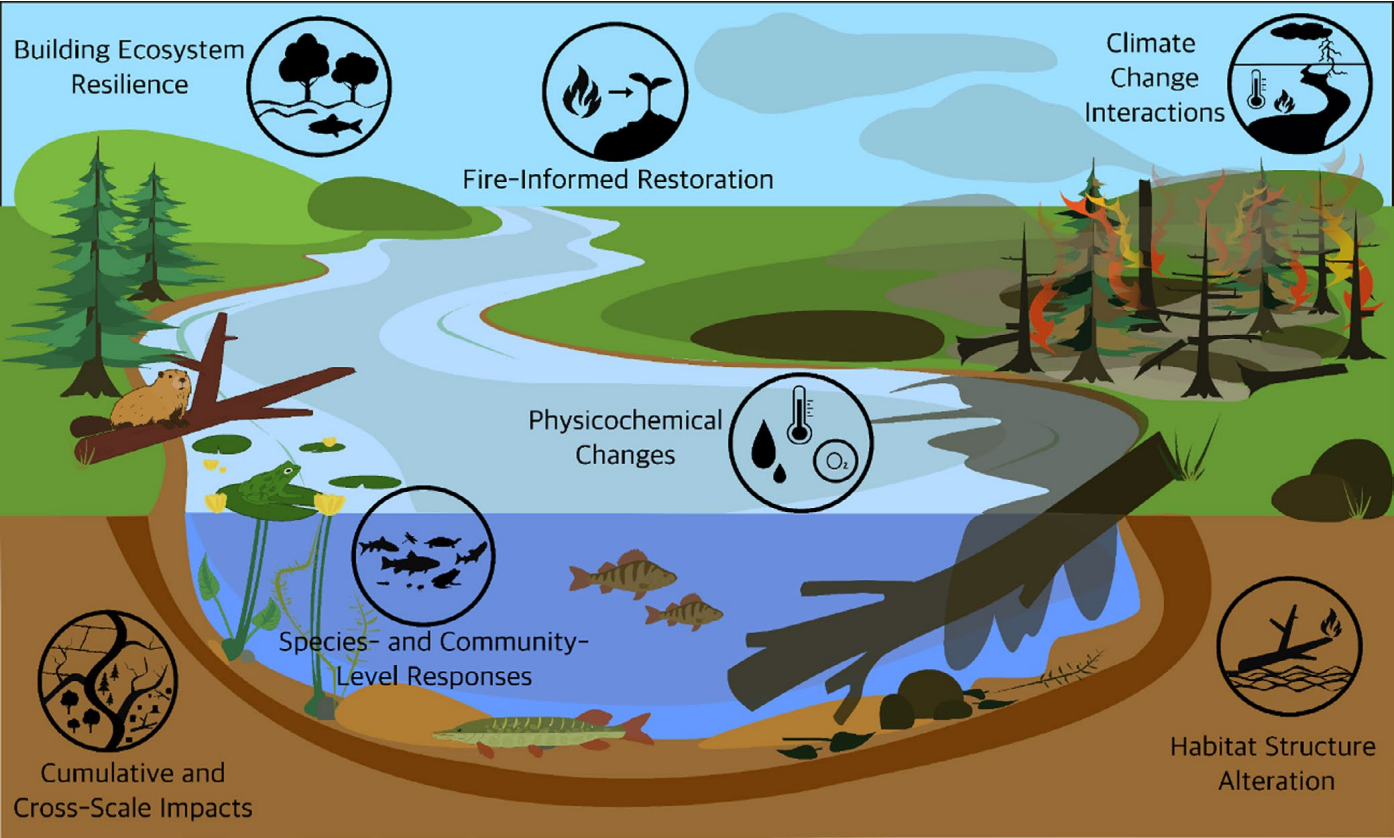
Key knowledge gaps to direct future research to further our understanding on the impacts of wildfire on freshwater ecosystems in North America.

**FIGURE 2:**
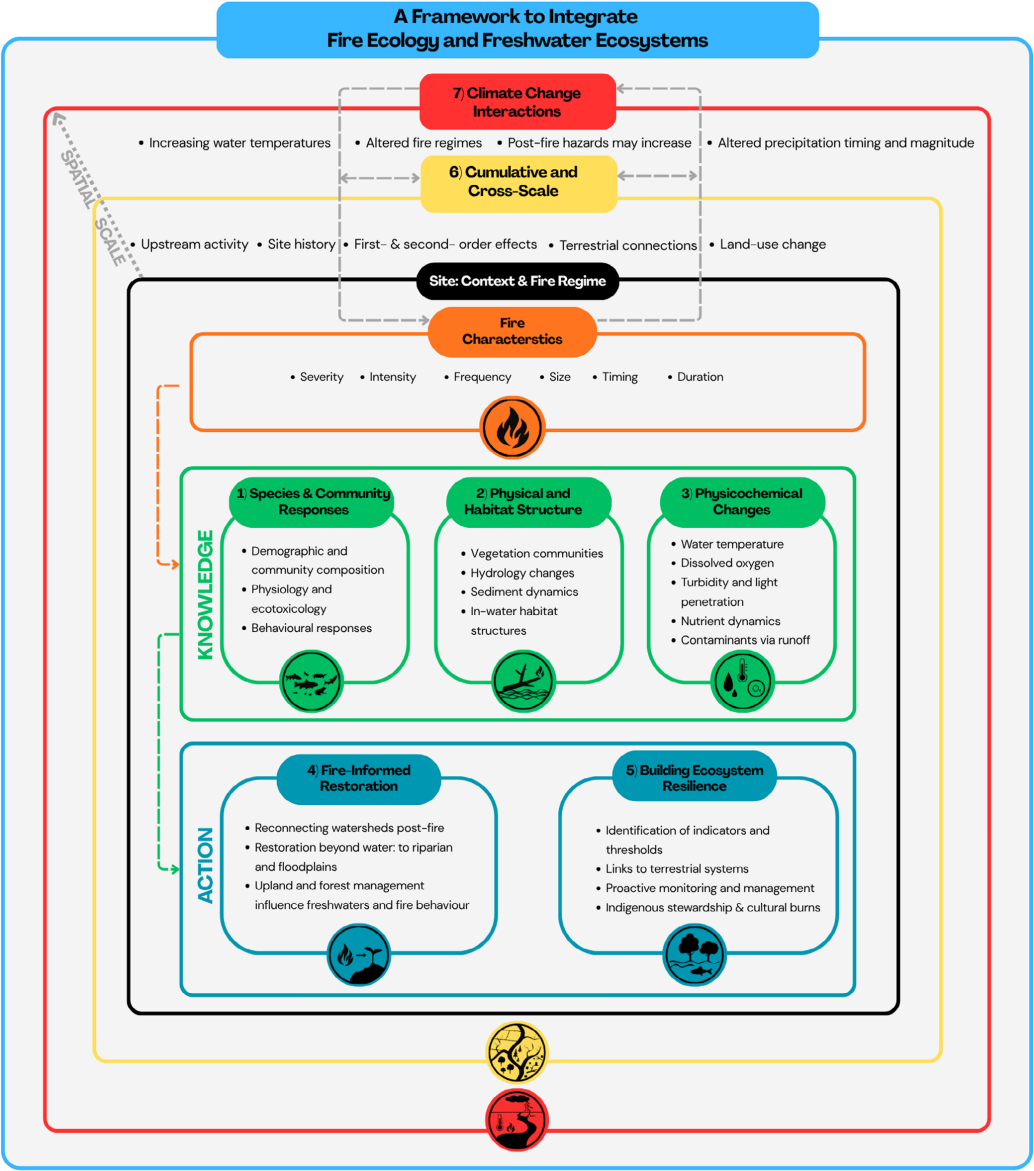
Conceptual framework illustrating key knowledge gaps at the intersection of fire ecology and freshwater ecosystems. Climate change (7) forms the overarching context, interacting with and often compounding cumulative and cross‐scale effects (6) across freshwater ecosystems. These broader processes, including land‐use change, site history, and upstream influences, shape local conditions and can both influence and be influenced by site‐level dynamics. At the site level, fire characteristics such as severity, intensity, frequency, size, and timing are embedded within this broader context. Importantly, feedbacks occur among climate change (7), cumulative and cross‐scale processes (6), and fire characteristics, creating a three‐way interaction in which each component can modify and reinforce the others across spatial and temporal scales. Within this framework, understanding wildfire characteristics is essential for predicting ecological responses. These fire attributes drive three primary ecological pathways within freshwater ecosystems: (1) species and community responses, (2) physical habitat and structural changes, and (3) physicochemical conditions, including temperature, sediment dynamics, and water chemistry. Together, these processes represent foundational knowledge required before translating fire–freshwater interactions into action, including (4) fire‐informed restoration and (5) strategies for building freshwater ecosystem resilience.

Below, we organize key knowledge gaps in the integration of fire ecology and freshwater ecosystems into thematic areas (Figure 1). For each knowledge gap theme (Figure 1), we briefly review the current state of the science and identify specific details contributing to the knowledge gap (see Table 2 for a summary of each gap). We also present a conceptual framework in which wildfire characteristics at the site level (Figure 2), including severity, intensity, frequency, size, and timing, shape freshwater ecosystems through three primary pathways: (1) species and community responses, (2) physical habitat and structural changes, and (3) physicochemical conditions. Understanding these pathways provides the foundation required to translate fire–freshwater interactions into action, such as (4) fire‐informed restoration and (5) strategies for building ecosystem resilience. Beyond the site scale, broader processes, including (6) cumulative and cross‐scale disturbances and (7) global climate change can also influence freshwater responses to wildfire (Figure 2).

**TABLE 2:** Summary of research topics within each identified knowledge gap on wildfire impacts to North American freshwater ecosystems, with examples illustrating potential research approaches.

Knowledge gap	Key research areas	Example	References
Species‐ and community‐level responses 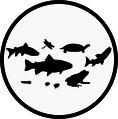	Understudied taxa including fish, macroinvertebrates, and mammals Sublethal impacts related to physiology and toxicology Response across severity gradients, management actions, and post‐wildfire precipitation	Before‐After Control‐Impact in Oregon, USA, found that after a severe wildfire, stream temperature, chlorophyll *a*, age‐0 cutthroat trout, and Pacific giant salamanders increased	Swartz and Warren (2022)
Physical and habitat structure alteration 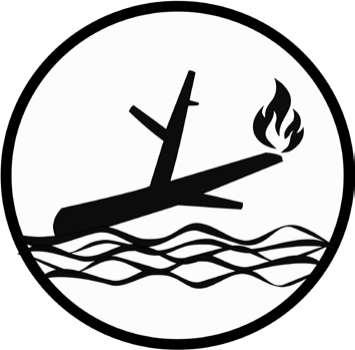	Impacts of wildfire and smoke on vegetation structure across freshwater habitat types Alterations to peat soil structure, function, and hydrological feedbacks	In Ontario Canada, depth of burn in peat across depths within rock barrens, where shallow peats were found to be more vulnerable to high burn severity	Wilkinson, Verkaik, et al. (2020), Wilkinson, Tekatch, et al. (2020)
Physicochemical changes 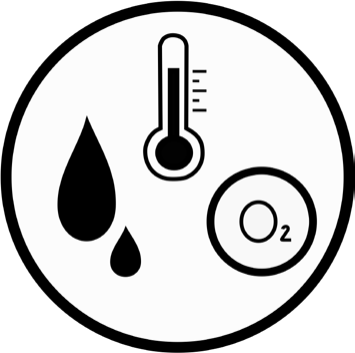	Extent of temperature related sublethal impacts to biota Dynamics of nutrient alterations associated with increased phosphorus, nitrogen, and cations Toxicological impacts from wildfire‐derived contaminants, with mobilization, bioavailability, and ecological effects shaped by pH shifts, sediment deposition, wetland retention, and burn severity Evolution of physiochemical properties and processes as ecosystems recover from wildfire	In the Northwest Territories, Canada, ash and char were incorporated into the peat matrix after a low‐severity wildfire, resulting in increased dissolved solutes, dissolved organic matter, and mercury concentrations	Ackley et al. (2021)
Fire‐informed restoration 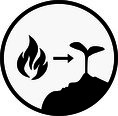	Response of biota to reconnection of freshwater ecosystems to floodplains to support recovery Implementation of riparian fuel and forest management to support recovery post‐fire	Before‐After Control‐Impact study in Idaho streams found prescribed fire did not produce the same impacts on periphyton, macroinvertebrates, amphibians, fish, or riparian and stream habitats as wildfire	Arkle and Pilliod (2010)
Building ecosystem resilience 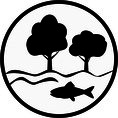	Identification of key indicators to assess resiliency of freshwater ecosystems to wildfire Identification of tipping points to inform management strategies Modulation of resilience by surrounding watershed and landscape‐level management strategies (e.g., cultural burning)	Before‐After Control‐Impact study in New Mexico streams found low resistance of insect taxa to postfire sediment‐laden floods, with moderate resilience of richness as recovery to pre‐fire levels occurred after four years, but community composition remained altered	Vieira et al. (2004)
Cumulative and cross‐scale impacts 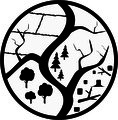	Complex direct and indirect fire effects influencing freshwater ecosystems that require near‐ and long‐term study to understand across diverse geographies, fire regimes, and ecosystems. Broad understanding of the fire ecology of freshwaters may come from careful integration of existing theories of systems dynamics from freshwater and terrestrial perspectives	Comparative study of fourth‐order streams affected by varied degrees of wildfire severity in the Western Cascades Oregon, U.S.A. Burns occurred in the summer of 2020. The first year of measurements was 2021, and it has continued annually (now at 2025). Measurements include vertebrate and invertebrate species responses, stream environment, riparian environments, wood recruitment, salvage logging, and age of forest stands	Coble et al. (2023)
Climate change interactions 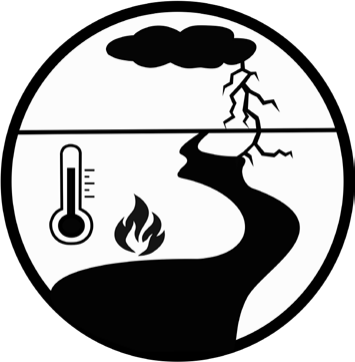	Compounding effects of climate change and wildfire, leading to more severe impacts on freshwater ecosystems Thermal stress on aquatic habitats and biota, particularly for ectothermic species Changes to snowpack, precipitation, and streamflow interacting with wildfire‐driven runoff and flood risk	In Washington, USA, the population vulnerability of bull trout was modelled to assess the impacts of simulated fire under climate and fire scenarios. The study determined that management of habitat connectivity and fire size can reduce climate change impacts	Falke et al. (2015)

### 3.1 Species‐ and Community‐Level Responses

Wildfire can both help and hinder freshwater diversity, depending most prominently on whether it is a natural or novel disturbance to the area, a distinction increasingly shaped by changing fire regimes. Wildfire can promote freshwater biodiversity by renewing riparian and freshwater habitats, thereby boosting productivity and complexity, or it can lead to severe effects that erode diversity and restructure channels and habitat (Bixby et al. 2015). Although most research has focused on the severe responses, in many cases, the effects of wildfire on freshwater biodiversity are subtle. Wildfires often burn patchily across instream and riparian areas of watersheds, potentially affecting a wide range of freshwater taxa, including fish (Gresswell 1999; Bisson et al. 2003; Dunham et al. 2007; Bixby et al. 2015; Curtis et al. 2025), amphibians (Russell et al. 1999; Bury et al. 2002; Pilliod et al. 2003; Dunham et al. 2007), reptiles (such as turtles; Russell et al. 1999), waterbirds (including geese and ducks; Saab and Powell 2005), mammals (including river dolphins and otters; Bowen et al. 2015; Aliaga‐Rossel et al. 2025), invertebrates (such as crabs, crayfish, mussels, and insects; Minshall 2003; Verkaik et al. 2015), phytoplankton (Paul et al. 2022), and freshwater and riparian plants (González‐Sargas et al. 2025; Marques et al. 2022). The effects of wildfire on flora and fauna include direct effects where the wildfire burns the organism by heat or gas causing injury or death. However, many effects of fire are indirect and can reduce riparian vegetation and alter the physical structure and function where the organism lives (more detail in next section; Whitney et al. 2015), including their food webs, which can be difficult to predict because fire affects freshwaters through multiple pathways, especially in watersheds with mixed burn severities (Roon et al. 2025). Generally, the greatest effects of fire are on those individuals, populations, or communities that are least mobile, such as stream algae (Klose et al. 2015), freshwater mussels (Lawrence et al. 2022), turtles (Steblaj et al. In Review), or species that are constrained to small geographic areas, such as gila trout ( *Oncorhynchus gilae* ). Over much longer time frames, wildfires can also modify species creating evolutionary adaptations. For example, Pacific salmon (*Oncorhynchus* spp.) have evolved strategies to survive large disturbances such as wildfires, including being sea‐run and mobile with flexible life‐histories (Waples et al. 2008), though local populations of a species may be vulnerable.

Globally, fish, macroinvertebrates (Rinne 1996; Rugenski and Minshall 2014; Martens et al. 2019), and primary producers, including stream algae are the most‐studied freshwater taxa following wildfire, and yet knowledge gaps persist for them and other understudied taxa. Fish population responses following fire are highly variable across the literature showing increases, decreases (from starvation, mortality, or emigration), and no apparent change (for richness, densities, and biomass). For example, native fish extirpations and population decreases can occur from landslides and channel reorganization events following precipitation events that follow wildfire though recolonization is rapid (< 2 years) if connectivity is maintained (Dunham et al. 2007; Erdozain et al. 2024). Another study on rainbow trout ( *Oncorhynchus mykiss* ) showed declines in burned streams likely because they were bioenergetically stressed as evidenced by their empty stomachs relative to trout in unburned streams (Beakes et al. 2014). Alternatively, sizes of young‐of‐year of coastal cutthroat ( *Oncorhynchus clarkii clarkii* ) and rainbow trout increased in streams with higher burn severity over unburned or lesser burned sites (Swartz and Warren 2022; Swartz et al. 2025) probably owing to increased habitat and bioenergetic success. These studies highlight differences in responses of freshwater fish to wildfire, underscoring the need to conduct further research to disentangle these dynamics. Community responses of macroinvertebrates or fishes include both changes to species composition for the majority of the studies or no change (Dunham et al. 2003; Minshall 2003; Malison and Baxter 2010; Cooper et al. 2021; Erdozain et al. 2024; Swartz et al. 2025). Mixed population and community responses are attributed to variability in post‐fire precipitation events, time lag between wildfire and sampling, fire severity, whether the riparian areas burned (Erdozain et al. 2024), and varying physiologies and life histories of fauna. Post‐fire forest harvest or salvage, a common management activity after fire, led to declines in tadpole density of coastal tailed frogs ( *Ascaphus truei* ), whose presence may have been complicated by metamorphosis (Swartz et al. 2025). Fire can also alter whole‐stream functions, such as nutrient spiraling, primary productivity, and ecosystem metabolism, creating cascading effects on stream food webs (Rugenski and Minshall 2014). Responses of primary producers generally show initial increases following fire (e.g., stream algae; Klose et al. 2015; peripython, Roon et al. 2025), though there are cases of no change. These ecosystem‐level responses often integrate multiple physical and biological pathways making them sensitive indicators of fire. Future work could evaluate the effects of wildfire on water quality factors and freshwater taxa across gradients of fire severity, post‐fire management actions, such as salvage and post‐fire precipitation events. In addition to studying population‐ and community‐level processes (e.g., Pleizier et al. 2025), future research could also examine how wildfires influence other biological processes, including behavior and physiology.

### 3.2 Physical and Habitat Structure Alteration

Wildfire alters the habitat structure of freshwater ecosystems in ways that differ among ecosystem types. The moderating influence of a high water table (Guêné‐Nanchen et al. 2022; Nelson et al. 2021; Mackay et al. 2024), physical peat properties (e.g., composition, depth, density), and hydrological fragmentation strongly influence the susceptibility of different types of freshwater ecosystems to wildfire (Crawford et al. 2024; Wilkinson, Verkaik, et al. 2020; Wilkinson, Tekatch, et al. 2020; Lukenbach et al. 2015). In both lotic and lentic ecosystems, the direct burning and loss of upland vegetation increases the risk of erosion and runoff facilitating sediment transport into freshwater and altering physical habitat through changes in turbidity, conductivity, nutrients, and contaminants (Gomez Isaza et al. 2022; McCullough et al. 2019; see section 3 for more details). These impacts can be exacerbated by high‐severity and high‐intensity fires that consume canopy cover (e.g., increased light availability potentially creating hotter and drier soil conditions, Legge et al. 2025, Kreye et al. 2018) and groundcover (e.g., loss of topsoil and associated nutrients/microbial communities), which can lead to long‐term or permanent changes (Ngole‐Jeme 2019; Certini 2005). In contrast, the loss of riparian vegetation along rivers and streams following wildfire is relatively well studied, particularly when riparian corridors burn at moderate to high severity or when fire extends directly to the stream edge (see Gomez Isaza et al. 2022; Erdozain et al. 2024; Bixby et al. 2015). This loss of riparian vegetation generally results in increased light availability and thus increased water temperature along a gradient of low‐ to high‐burn severity (River et al. 2026), which alters thermal habitat conditions for freshwater species and can deplete dissolved oxygen (DO) content. Wildfire also generally increases stream flow and peak flows, resulting in increased sediment and debris flows, which can be a major source of carbon, nutrient, and contaminant inputs to freshwaters post‐fire, that negatively alter habitat for phytoplankton and macroinvertebrates in the first few years after fire (Paul et al. 2022).

Although lakes may have similar responses to loss of riparian vegetation as lotic systems, such as increased inputs from the watershed, combined effects of fire, increasing air temperatures, and other concurrent disturbances (e.g., logging) make it difficult to disentangle the drivers of observed water temperature increases (McCullough et al. 2019). Despite the possibility of streams propagating wildfire effects to downstream lakes, lake volume and water residence time are likely the primary factors mediating how lakes respond to fire (McCullough et al. 2019). In one lake case study, prolonged wildfire smoke reduced light availability which altered thermal structure and primary productivity, leading to modest shifts in zooplankton community composition, while zooplankton biomass and migration patterns remained unchanged (Scordo et al. 2021). However, such responses are likely to vary with lake morphometry, trophic status, mixing regime, background climate conditions, and the duration and intensity of smoke exposure, thereby highlighting the importance of research across diverse freshwater ecosystem types and settings.

Wetlands may experience disproportionate physical alteration due to their susceptibility to higher wildfire severity, which can stem from the ephemeral nature, shallow depths, and brief hydroperiods of some wetland types (Kominoski et al. 2022; Wilkinson, Verkaik, et al. 2020; Wilkinson, Tekatch, et al. 2020). Wildfire can alter wetland habitat structure and function in ways that are highly variable and influenced by regional geographic context. Fire‐induced reductions in water storage may compromise the suitability of wetlands as habitat for semi‐aquatic taxa, including turtles, frogs, and salamanders (e.g., Roberts et al. 2023; Gould et al. 2022; Struecker et al. 2021); however, wildfire may also create novel freshwater microhabitats through ephemeral ponding suitable for semi‐aquatic species within 2.5 years since fire (Delay et al. 2024). High vegetation and soil burn severity can alter habitat structure and function for snakes whose survival is tightly coupled with wetland thermal and hydrological dynamics (North et al. 2024; Markle, Wilkinson, and Waddington 2020) and frogs whose breeding persistence is linked to percentage of unburned habitat surrounding ephemeral wetlands (Bailey et al. 2025). Wetlands with interspersed patches of open water (e.g., marsh, ponds, fen) typically experience lower fire severities and therefore decreased habitat alterations due to limited fuel continuity, high soil moisture content, and the presence of beaver dams (Markle et al. 2022; Fairfax and Whittle 2020). In cases of lower burn severity, revegetation is generally possible if there is adequate water availability; however, in drier conditions, regeneration of wetland vegetation may not occur (Mackay et al. 2024), and wetlands could transition towards other ecosystem types (e.g., open fens to shrubby fens; Guêné‐Nanchen et al. 2022).

Deep peatlands generally recover quickly and remain resilient to wildfire (Wilkinson, Tekatch, et al. 2020) due to feedbacks that maintain a high water table (Waddington et al. 2015; Furukawa et al. 2025), although vulnerability to severe burning can vary based on vegetation structure (i.e., presence of trees; Johnston et al. 2015) and peat properties (e.g., depth, density; Benscoter et al. 2011). While physical and chemical shifts in peat properties and microbial disruptions are frequently observed following fire, with the magnitude of change influenced by burn severity and the depth of peat consumption (e.g., Allingham et al. 2024; Ackley et al. 2021; Flanagan et al. 2020; Wu et al. 2020), shallow peatlands may operate near critical ecohydrological thresholds because of their relatively limited capacity to regulate hydrological conditions (Furukawa et al. 2025; Sutton et al. 2025). This makes them disproportionately vulnerable to disturbances such as increased fire severity and frequency in the context of climate change (McCarter et al. 2024; Sutton et al. 2025). In fire‐disturbed peatlands, drainage combined with deep smoldering fire may generate altered hydrological regimes that facilitate invasive species spread, such as the common reed ( *Phragmites australis* ; Link et al. 2024), which negatively impacts habitat structure by homogenizing bird communities (Robichaud and Rooney 2022) and altering turtle habitat selection (Markle and Chow‐Fraser 2018). Other types of wetland ecosystems, such as swamps, were found to experience similar habitat alterations due to wildfire. Mesocosm experiments have suggested that in groundwater‐dependent upland swamps, wildfire may reduce vegetation species richness and cause shifts in community composition, with hydrological change and fire acting synergistically to drive transitions toward drier conditions (Mason et al. 2023).

While hydrological and vegetation regime shifts may weaken the resilience of freshwater ecosystems to fire, how these processes interact with changing hydrological dynamics, climate variability, and fire‐related feedbacks to drive potential irreversible changes to habitat structure and function remains poorly understood (Mason et al. 2023; Link et al. 2024). Broadly, the effects of altered fire regimes on vegetation structure of lakes and wetlands (e.g., marshes, swamps, coastal wetlands) also remain an important knowledge gap (Bixby et al. 2015). In particular, uncertainty persists around the impacts of wildfire in open peatlands, especially as climate change and associated drying could shift vegetation composition (Guêné‐Nanchen et al. 2022). Furthermore, there is also a lack of research on the indirect impacts of wildfire on vegetation structure. For example, wildfire smoke is known to influence light and thermal regimes, and productivity of lakes (Scordo et al. 2021; Farruggia et al. 2024; Smits et al. 2024), but there is little research on how this translates to wetland or riparian vegetation growth. For peatland environments, critical gaps remain in understanding the long‐term resilience and recovery of peat soil structure and function, particularly across peat depths and under repeated wildfire exposure (McCarter et al. 2024; Sutton et al. 2025).

### 3.3 Physicochemical Changes to Freshwater Environments

Wildfires impact the physicochemical conditions in many surface freshwaters (e.g., wetlands, lotic systems, lakes) over varying timescales, where short‐term impacts can have potentially acute effects on freshwater biological communities but longer‐term changes to water chemistry are often observed with less certain impacts to ecosystems and communities (Bixby et al. 2015; Curtis et al. 2025; Sánchez‐García et al. 2023; Silins et al. 2014).

Initially, the loss of riparian and shoreline vegetation around freshwaters leads to an increase in sunlight reaching the water surface and an increase in water temperature in both lotic (e.g., streams, Cooper et al. 2015; Paul et al. 2022; Sanders et al. 2022) and lentic ecosystems (Paul et al. 2022). Increases in surface water temperatures are greater with higher fire severity, lower water volumes (less thermal mass), and slower water speeds (e.g., meandering rivers, shallow lakes; Burton 2005; Beakes et al. 2014; Koontz et al. 2018). Elevated surface water temperatures can persist for years to decades as the surrounding upland vegetation recovers from the wildfire (Burton 2005; Koontz et al. 2018); however, it is rare that temperatures increase beyond the ecosystems' thermal boundaries (Bixby et al. 2015; Gomez Isaza et al. 2022). Nonetheless, sublethal thermal impacts to temperature sensitive taxa, such as reptiles, amphibians, and fish, can directly alter metabolisms and life history traits (Beakes et al. 2014; Rosenberger et al. 2015; Arroyo‐Morales et al. 2023). Importantly, the increase in stream surface water temperature decreases the solubility of oxygen gas (O_2_), contributing to mass mortality events (Burton 2005). Temperature increases can also stimulate other biogeochemical processes that often have greater detrimental impacts to freshwater ecosystems when combined with other wildfire impacts (Gomez Isaza et al. 2022).

Wildfires can also result in alterations to nutrient dynamics within lentic and lotic ecosystems. For instance, smoke and ash from wildfires can result in temporary increases in bioavailable phosphorus and nitrogen species in surface waters of lakes (Olson et al. 2023) and streams (Rhoades et al. 2025). Precipitation following wildfires, or sometimes even during wildfires (Curtis et al. 2025; Rhoades et al. 2025) can result in the flushing of both water soluble and particulate (i.e., fire destabilized soils) inorganic nutrients and some cations (i.e., calcium and potassium) into surface waters (Bixby et al. 2015; Bladon et al. 2014; Brown et al. 2015; Emmerton et al. 2020). The timing and magnitude of these flushing events varies based on catchment characteristics, precipitation patterns, and other landscape disturbances (e.g., Paul et al. 2022). Regardless of the differences in delivery, increased nutrients and energy inputs post‐wildfire in freshwater ecosystems can lead to eutrophication due to increased algae production and microbial activity (Paul et al. 2022; Sánchez‐García et al. 2023). However, response in primary production as a result of fire‐induced biogeochemical changes (“pyroeutrophication”, Waters et al. 2023) is not consistent (e.g., Earl and Blinn 2003; Harris et al. 2015), suggesting that burn intensity is a mediating mechanism (Waters et al. 2023). Increases in algae observed, coupled with increased water temperatures, can lower DO in the water column of both impacted lentic and lotic freshwater ecosystems, which has resulted in mass mortality events shortly after wildfires (Burton 2005; Gomez Isaza et al. 2022). Understanding the magnitude, directionality, and timescales of these compounding wildfire impacts on both physicochemical environments and biological systems remains unresolved.

Runoff during and after wildfires transports a range of contaminants, including potentially toxic elements (PTEs), polycyclic aromatic hydrocarbons (PAHs), organic compounds, and fire‐fighting chemicals (Campos et al. 2015; Campos and Abrantes 2021; Murphy et al. 2020, 2025; Nunes et al. 2017; Pennino et al. 2022; Puglis et al. 2022; Schäfer et al. 2010; Wu et al. 2022). Post‐fire shifts in water chemistry, particularly elevated pH, influence contaminant solubility and speciation, altering their mobility and bioavailability (Bixby et al. 2015; Emelko et al. 2011; Murphy et al. 2020, 2025; Rust et al. 2019). Mobilization of PTEs can occur shortly after fire and persist for years (Smith et al. 2011; Paul et al. 2022), but is usually below acute thresholds unless legacy contaminants are remobilized. Yet, shorter fire return intervals may increase contaminant accumulation in sediments or enhance methylmercury bioaccumulation (Ackley et al. 2021; Bladon et al. 2014; Gomez Isaza et al. 2022). Unlike PTEs, PAHs, and firefighting chemicals have been detected at harmful concentrations for aquatic organisms (Gomez Isaza et al. 2022; Morrison et al. 2025). However, most toxicological evidence is based on organism‐level laboratory or mesocosm studies, leaving ecosystem‐scale consequences unresolved (Gomez Isaza et al. 2022). An additional impact associated with the application of aerial fire retardants is the delivery of high concentrations of phosphorus‐ and nitrogen‐based compounds to freshwater ecosystems, often at rates exceeding those typical of agricultural nutrient applications (Moore et al. 2025). Excessive nutrient loading to surface waters can endanger aquatic life by stimulating harmful algal blooms, increasing microbial respiration, reducing dissolved oxygen concentrations, and altering community composition (Moore et al. 2025).

Wetlands play a central role in regulating post‐fire contaminants through influencing water and chemical fluxes (Bixby et al. 2015; Casey and Klaine 2001; Hefting et al. 2006). Unburned wetlands buffer downstream impacts by retaining particulates, transforming nutrients, and sequestering contaminants via adsorption, degradation, or plant uptake (Kominoski et al. 2022; McCarter et al. 2024; Zhao et al. 2021). When wetlands, chiefly organic soil rich peatlands, themselves burn, outcomes depend strongly on fire severity and extent (Ackley et al. 2021; Li et al. 2023; McCarter et al. 2024; Wu et al. 2022). Combustion of peat and other organic soils creates hydrophobic layers that reduce infiltration, enhance runoff, and deliver pulses of sediment and associated contaminants to receiving waters, typically during rainfall events shortly after wildfires (Chanasyk et al. 2003; Elmes et al. 2019; Kettridge et al. 2014; Marcotte et al. 2024, 2025). Burned organic soil wetlands often show elevated pore‐water nutrients and contaminants, with chemical profiles shaped by fire temperature (Abraham et al. 2017; Li et al. 2023; Sánchez‐García et al. 2023). The ecological significance of these changes is mediated by shifting ecohydrological connectivity between soil pore, wetland vegetation, and surface waters, which can delay the delivery of dissolved constituents, such as PTEs, for months or longer (Emelko et al. 2011; Richardson et al. 2024; Rust et al. 2019). Such temporal lags complicate detection of biological impacts, underscoring the need for longer‐term studies that capture both immediate and delayed wildfire effects on freshwater ecosystems. In addition, the combined effects of catchment and wetland ecohydrological recovery and biogeochemical change (i.e., landscape connectivity) on freshwater organisms remain poorly understood.

### 3.4 Fire‐Informed Restoration

Historically, functional freshwater ecosystems (lotic and lentic) interacted with disturbance processes such as wildfire, floods, and landslides, creating dynamic freshwater landscapes characterized by mosaics of habitat quality and quantity for aquatic biodiversity over time (Hessburg et al. 2016). Wildfire suppression, coupled with warmer and drier landscape conditions, has altered the processes that supported native ecosystems. Additionally, wildfire suppression has changed fuel availability, which affects wildfire severity and burn intervals in both upslope (Agee and Skinner 2005) and riparian areas (Dwire et al. 2016). In many regions, these altered fire regimes also reflect the systematic suppression of Indigenous cultural burning practices that historically contributed to landscape heterogeneity and ecosystem function across terrestrial and adjacent freshwater environments (Kimmerer and Lake 2001; Hoffman et al. 2021). As managers seek to recover imperiled freshwater species, restoration that prepares freshwater ecosystems to take advantage of fire may offer a means for natural rejuvenation. However, freshwater ecosystem restoration generally ignores the role of wildfire in contributing to their habitat complexity. Implementing freshwater restoration that facilitates the connectivity of nutrients, sediments, and large wood from hillside and riparian environments with valley‐floor rivers, wetlands, and ponds has potential to recover processes that naturally support freshwater ecosystems (Beechie et al. 2010). For example, floodplain reconnection is hypothesized to provide refugia to native species during wildfire, and accumulate post‐wildfire sediments (Pugh et al. 2022). Similarly, restoration of beaver or beaver‐style dams has been shown to recover wetlands, rewetting valleys and changing how fire moves across the landscape (Fairfax and Whittle 2020). More investigation is needed of the effectiveness of reconnecting rivers, wetlands, and ponds to their floodplains and riparian areas in order to design and identify restoration locations that are intended to capitalize on future wildfire to enhance habitat quality and composition. Additionally, the context of historic wildfire regime and current forest vegetation age and composition contribute to diverse outcomes of coupled floodplain‐upslope restoration. These relationships require research that spans wildfire regimes and forest types to effectively understand the effects of restoration and management for the diversity of freshwater environments (e.g., lotic and lentic).

Different forest management practices that facilitate recovery of watershed‐scale processes connecting upslope and riparian areas are needed, which are customized by local patterns of topography, climate, soil composition, forest stand structure, local wildfire regime, and river network configuration. Thus, forest management must be customized to local forest composition and wildfire regimes. For example, dry forests generally burn more frequently than mesic forests, and their fire severity and ignition potential are often linked with fuel loading. Therefore, management of these forests includes reduction in forest fuels and thinning of canopies (see Agee and Skinner 2005). These actions have not generally included riparian or wetland areas (Dwire et al. 2016), and the outcomes of the actions in riparian areas are not well understood. For example, Arkle and Pilliod (2010) evaluated prescribed fire in riparian areas and found that it may not result in the natural subsidies of nutrients and material to freshwater food webs that are typically associated with wildfires. Post‐fire restoration techniques for peatlands are currently being developed to increase the density of wetland on the landscape post fire (Gage et al. 2024). Thus, additional experimentation to evaluate treatment effectiveness and outcomes is necessary in riparian and wetland areas in both dry and mesic environments.

Active forest management in mesic forests could also facilitate a shift towards coupled wildfire‐related upslope and riparian management. Modification of tree composition in both upslope and riparian areas away from uniform stands of similarly flammable conifers, towards greater stand diversity, could facilitate ecological recovery (Hessburg et al. 2022), which has been conducted within the USA with aspens (Harris et al. 2025; Krasnow et al. 2012). Research demonstrates the important role of non‐stand‐replacing wildfires in old‐growth moist‐wet forests (Tepley et al. 2013; Merschel et al. 2024); however, prescribed fires are not generally conducted in moist‐wet forests (Reilly et al. 2022). Rather, incorporating diversified stand composition by tree age and species in riparian and upslope environments may be the most tractable way to recreate forest complexity that contributes to the development of burn mosaics, and forest regeneration after wildfire that benefits upslope stands and embedded riparian areas and associated lentic and lotic habitats. Thus, active management to facilitate the functional expression of wildfire at forest scales will differ across dry and mesic environments, reflecting variability in forest composition, drivers of fire severity, and return intervals.

### 3.5 Building Ecosystem Resilience

Ecological resilience, termed by Holling (1973), refers to the amount of disturbance that an ecosystem can withstand while maintaining its original function. Inherently, ecological resilience is a measure of resistance to change under stress (resistance) and the ability to recover (resilience; Connell and Sousa 1983). Here we encompass the two using the definition of resilience by Scheffer (2009) as ‘the ability to absorb disturbances and re‐organize under change to maintain similar functioning and structure’. Resilience is deemed to be overstepped when an indicator such as community composition or nutrient concentration does not reflect the original ecosystem (or control site), suggesting a regime shift or transition to an alternate stable state has occurred (Scheffer 2001; Carpenter et al. 2003). Here, key indicators are used as proxies for ecological function, but due to the diverse functions of freshwater ecosystems, multiple measures of function will likely be required to be integrated to effectively evaluate resilience (Grantham et al. 2019; Jaiswal et al. 2021). Further, Indigenous Nations and Peoples are likely to hold their own Knowledge and understandings of ecological balance that may differ from those determined through Western science and can be applied to identify thresholds for building resilience (Grenz 2025, 145).

The resilience concept has been applied to evaluate many other disturbances in freshwater ecosystems, including nutrient input to shallow lakes (Scheffer and Jeppesen 2007), the impacts of large water infrastructure projects on rivers and streams (Grantham et al. 2019), and the climate change impacts of changing flow regimes and higher severity floods and droughts on freshwater ecosystems (Aldous et al. 2011). Most studies use a number of indicators specific to their metric of interest (e.g., flow rate, nutrient concentration, water temperature, population density, community composition) to measure and evaluate resilience, making attempts at cross‐comparison between studies or disturbances challenging. With particular focus on wildfire, Lewis et al. (2014) assessed the impacts of natural wildfire on boreal lakes and found high resistance and resilience (i.e., no significant change from pre‐disturbance conditions) in most trophic levels. Conversely, Vieira et al. (2004) found low resistance of insect taxa to post‐fire sediment‐laden floods with moderate resilience of richness shown by recovery to pre‐fire levels after four years. Further, ecohydrological thresholds related to tree basal area and peat depth have been used to identify tipping points in ecosystem resilience to wildfire in inland wetlands (Wilkinson, Moore, Flannigan, et al. 2018; Wilkinson, Tekatch, et al. 2020). These thresholds have been applied to inform management strategies related to fire severity, carbon sequestration, and snake and turtle species‐at‐risk habitat (see Markle, Moore, and Waddington 2020; North et al. 2024) by identifying areas of higher impact (lower resistance) on the landscape. While some thresholds have been identified in inland wetlands and peatlands, it is likely that other freshwater ecosystems have easy‐to‐measure thresholds that represent tipping points for increasingly severe fire impacts; however, this represents a knowledge gap in many systems, largely due to a lack of accurate pre‐ and post‐fire data.

Thresholds to cascading disturbances are common for hydrogeomorphic processes such as flooding, landslides, and debris flows. Infiltration‐excess runoff and runoff‐generated debris flows after wildfire are both dependent on the exceedance of rainfall intensity thresholds (Cannon et al. 2011; Staley et al. 2013) and can have significant impacts on freshwater ecological systems, particularly where wildfire‐induced soil water repellency (hydrophobicity) reduces infiltration and increases overland flow (Caltabellotta et al. 2022). These threshold processes will affect ecosystems differently in relation to their hydrological dynamics with greater impacts for ecosystems receiving water from large basins and/or steeper slopes (Staley et al. 2017). While wildfire resilience research is growing, there remains a sparsity of research regarding both process‐based resilience thresholds and impact‐based thresholds of plant, animal, and microbial communities across the continuum of freshwater ecosystems.

The resilience of freshwater ecosystems to wildfire is connected to, and somewhat dependent on, the resilience of the surrounding terrestrial ecosystems. As such, the resilience of surrounding terrestrial ecosystems will moderate the magnitude and duration of wildfire impacts on freshwater ecosystems. For example, ecosystems that are hydrologically connected to burned uplands will be impacted based on the burn severity of those uplands, where higher severity wildfire may cause changes to water and sediment fluxes, water quality, biomass, and both sedimentation and energy exchanges in downstream lakes, ponds, or wetlands, as well as changes to the ecosystem itself, such as combustion of vegetation. The magnitude of such impacts, particularly those downstream, will also be modulated by the degree of ecological, hydrological, and sediment connectivity across the landscape (Grantham et al. 2019; Murphy et al. 2019). Further, many post‐fire hydrogeomorphic processes (e.g., flooding, debris flows, landslides) are threshold processes that are often associated with a time‐lagged response to the fire itself. Thus, given that post‐fire floods and debris flows can represent some of the most significant modifiers of and disturbances for in‐stream and wetland habitat (as well as downstream, i.e., lacustrine habitat), there may be implicit threshold behavior involved in when, and under what conditions, freshwater ecosystems and populations are pushed beyond the limits of their resilience. The resistance and the resilience of the freshwater ecosystem will then determine the amount of change incurred. Hence, managing for increased resilience in freshwater ecosystems requires a watershed approach where the spatial and temporal connectivity of landscape units is currently a significant knowledge gap.

Decades of research aimed at protecting forest resources have informed efforts to manage forested ecosystems for increased resilience, leading to the development of fuel‐reduction treatments to dampen the effects of fire (Agee and Skinner 2005). There are many ecozone, forest‐type, or jurisdictional boundary‐specific guides and plans where fuel treatments are sometimes referred to as wildfire risk reduction treatments (BCWS 2024). Although many regional plans reference fuel treatments or wildfire risk reduction treatments (BCWS 2024), in practice very little fuel reduction work is implemented, as seen in British Columbia, Canada, where only limited areas have been treated, and there has been far greater spending on suppression than prevention (Daniels et al. 2024). The principles of forest resilience could be followed for proactive management around freshwater ecosystems in order to reduce the magnitude of the impacts on them (as described above). Further, treatments could be conducted in treed freshwater ecosystems to reduce fire impacts. Research in this space is limited, but see Wilkinson, Moore, Thompson, et al. (2018) and Thompson et al. (2020). Principles for fire‐resistant forests in western North American forests include retaining and encouraging (large‐diameter) fire‐resistant trees, as well as reducing surface fuels, ladder fuels (also referred to as increasing canopy base height or height to live crown; see Table 1 for glossary), and crown fuel load (Agee and Skinner 2005). While these principles are typically achieved using heavy machinery, alternative implementations should be considered to protect the structure and function of freshwater ecosystems.

Fuel treatments are often confined to the immediate vicinity surrounding communities or critical infrastructure, known as the wildland‐urban interface. This approach does not easily scale to provide a watershed scale strategy. Indigenous land and fire stewardship, may be more appropriate to treat large areas across watersheds. Prescribed and cultural fire (part of Indigenous fire stewardship) utilize fire on the landscape in intentional ways, often during shoulder or winter seasons when fire impacts are reduced. Western prescribed burns can reduce surface and ladder fuel loads and may provide additional ecological benefits in fire‐prone landscapes (Arkle et al. 2012). Indigenous‐led cultural burning similarly represents a low‐impact, place‐based approach that enhances ecosystem diversity, supports the management of complex socio‐ecological resources, and reduces wildfire risk, thereby contributing directly to ecosystem resilience; however, systemic and colonial barriers continue to limit the re‐engagement of Indigenous fire stewardship despite millennia of practice (Hoffman, Christianson, Dickson‐Hoyle, et al. 2022). Together, these approaches support resilience in forested ecosystems by reducing the likelihood of high energy (high intensity) wildfire, where intensity is correlated with severity (i.e., impacts; Agee and Skinner 2005). However, research on the efficacy of upland/adjacent fuel management treatments on the resilience of freshwater ecosystems to wildfire is severely lacking, as are studies on fuel management within treed freshwater ecosystems (but see Wilkinson, Moore, Thompson, et al. 2018 and Thompson et al. 2020) and cultural burn effects on freshwater ecosystems. Challenged by the probability of fuel treatments, wildfire, and research funding coinciding, fuel‐treated stands that are in or adjacent to freshwater ecosystems should be actively considered for prescribed or experimental fires, reduction of barriers preventing cultural burning practices, and targeted post‐fire monitoring.

Proactive management strategies may include mapping ecosystem and watershed vulnerability to (severe) wildfire and post‐fire natural hazards (Markle et al. 2022; Wilkinson et al. 2021; Cafferata et al. 2021), identifying ecological refugia (Tekatch et al. 2025) and critical co‐occurrence habitat across landscapes (Markle, Moore, and Waddington 2020), and mitigating other ecosystem stressors (e.g., land‐use change such as forestry). Reducing other ecosystem stressors is critical, as evidence from varied ecosystems and disturbances suggests that ecosystem health before disturbance has greater or equal importance on the outcome of disturbance (recovery vs. non‐recovery) than the characteristics and magnitude of the disturbance itself. This is likely rooted in the ability of healthy landscapes and ecosystems (e.g., free of invasive plants, low levels of pollutants) to resist severe wildfire impacts by maintaining typical moist to wet conditions (e.g., via the presence of beaver dams; Fairfax and Whittle 2020), and to reorganize and recover using typical seedbank and resprouting mechanisms.

### 3.6 Cumulative and Cross‐Scale Impacts

Fire events are one of many disturbances that overlap and interact over time, resulting in the cumulative impacts of fire for a landscape or watershed. For example, post‐fire salvage logging results in changes to standing and downed wood legacies (Lindenmayer et al. 2008; see Table 1 for glossary) that can influence wood recruitment to freshwaters (Reeves et al. 2006). However, the broader cumulative impacts of post‐fire salvage on freshwater communities and conditions will vary depending on context, including time since fire, stand conditions at the time of fire (e.g., structure and composition of the stand), the location and type of salvage (e.g., topographic slope, salvage method, amount of wood removed, implementation of erosion control, upstream/downstream burn severity), and the ecological response of interest (Karr et al. 2004; Reeves et al. 2006; Lindenmayer et al. 2008). These diverse outcomes require further study and are a clear example of the need to understand the “it depends” of fire and disturbance ecology in freshwater ecosystems.

In fire ecology, we characterize a continuum of first‐order fire effects as the direct or immediate influences from fire and second‐order fire effects (see Table 1 for glossary) as the indirect, longer‐term outcomes of a wildfire. The direct and indirect effects are inherently cumulative. For example, fire‐induced soil alteration and the development or enhancement of soil water repellency can increase runoff and soil erosion while reducing soil‐water infiltration (DeBano 1981; Doerr et al. 2000; Parsons et al. 2010). These post‐fire hydrological changes can, in turn, affect sediment loads, water chemistry, and the morphology of freshwater systems, particularly in streams (Moody and Martin 2009; Eaton et al. 2010; Rust et al. 2018; Ridgway et al. 2024). In some cases, post‐fire natural hazards such as debris flows or landslides may deliver large volumes of sediment and woody debris to streams, depending on the magnitude and timing of rainfall events following the fire (Ridgway et al. 2024). Furthermore, restoration and land management activities implemented after fire, including erosion control treatments and salvage logging, can influence both the biological and physical properties of stream ecosystems. Reductions in riparian canopy cover can increase stream temperatures by exposing channels to direct sunlight (Beakes et al. 2014), while subsequent disturbances such as windthrow or insect outbreaks may further alter forests adjacent to aquatic habitats. Collectively, these processes interact in complex ways, and the added influence of climate change introduces further uncertainty into how post‐fire landscapes will affect freshwater ecosystems.

While our focus has been to highlight cumulative impacts that may affect freshwaters after a given fire event, it is also important to consider that every watershed has a pre‐fire history of disturbance (i.e., site history; Figure 2), with landscape memory and legacies of those overlapping events (Peterson 2002). For example, repeat, short‐interval fire events (i.e., ‘re‐burns’), whether from wildfire, prescribed fire, or cultural burning, contribute to the nature and degree of cumulative impacts and need further study from ecological and hydrogeomorphological perspectives. Additionally, insect outbreaks (Talucci et al. 2020), wetland drainage (Wilkinson, Moore, Flannigan, et al. 2018; Wilkinson, Verkaik, et al. 2020; Wilkinson, Tekatch, et al. 2020), and restoration (McCarter et al. 2021), or forest harvest (Zald and Dunn 2018) before a fire event can fundamentally influence wildfire effects, contributing to the post‐fire conditions and responses. There are complex direct and indirect pathways in which the effects from fire may influence freshwater ecosystems that require near‐ and long‐term study to understand across diverse geographies, fire regimes, and ecosystems. Ultimately, it is important to recognize that while wildfire can represent a severe disturbance to freshwater ecosystems that, particularly in the short‐term, may reduce biodiversity and ecosystem function, it can also be a critical process for the renewal and rejuvenation of riparian and freshwater habitats that may boost productivity and ecosystem complexity in the long‐term (e.g., Pugh et al. 2022). Recognition of a historical range of variability of characteristic fire effects and fire regimes for different freshwater ecosystems, and distinguishing them from uncharacteristic effects of shifting fire regimes is critical for development of freshwater fire ecology.

Freshwater ecosystems are structured by connections across multiple spatial and temporal scales that influence their response to fire events. For example, high severity fire in the riparian zone of narrower first‐ or second‐order streams may strongly influence the local in‐stream environment with increased light and temperatures (e.g., Beakes et al. 2014), but will likely have more varied or limited effects on stream conditions in wider third‐ or larger order streams (e.g., Coble et al. 2023). Whether or not fire effects from upstream are translated to downstream environments will depend first and foremost on the hydrogeomorphic connectivity throughout the watershed (e.g., Murphy et al. 2019). In well‐connected watersheds though, the area of the watershed, the percentage of upstream area burned, and the severity of the wildfire will all be important factors influencing the magnitude of downstream effects, which would have relatively quick impacts. Understanding the degree to which upstream fire effects influence freshwater response timing and magnitude relative to local fire effects (e.g., burn of adjacent riparian forest) is an important yet open question that needs further research. Broad understanding of the fire ecology of freshwaters may come from careful integration of stream ecology's river continuum concept (Vannote et al. 1980) that suggests predictable features of aquatic ecosystems from upstream to downstream, along with the process domains concept (Montgomery 1999) that integrates geomorphology and disturbances into our understanding of watershed dynamics, and landscape ecology of terrestrial ecosystems (Turner 1989) that considers the importance of spatial heterogeneity and both local and landscape context. In addition to lotic systems (e.g., streams and rivers), extending these frameworks to lentic habitats (e.g., ponds and lakes) by accounting for factors such as basin morphology, hydrological connectivity, and water residence time, as well as system‐specific cumulative stressors such as nutrient loading, urbanization, water level regulation, and invasive species that influence how fire‐driven inputs are processed within these systems.

### 3.7 Climate Change Interactions

Now and into the future, an increasingly important consideration is how ongoing climate change could alter the impacts of wildfire on freshwater ecosystems. Climate change could exacerbate or alter the impacts of wildfire in different ways. First, as mentioned previously, climate change is altering the severity, extent, and frequency of wildfires. However, projected changes in fire regimes are highly ecosystem‐dependent, and projected changes in fire severity and return intervals differ across North American biomes. For example, boreal forests are expected to experience shorter return intervals and more frequent high‐severity fires (Buma et al. 2022; Palm et al. 2022; Walker et al. 2023), while western temperate and montane forests may see increased fire severity due to warming, drought, and fuel aridity (Abatzoglou et al. 2017; Wasserman and Mueller 2023). Historically low‐fire systems, including some coastal and wetland‐dominated landscapes, may experience increased vulnerability (Nelson et al. 2021) and novel fire activity (e.g., Garcia et al. 2021). Accordingly, the impacts of any given ignition event (e.g., lightning strike) on freshwater ecosystems may be magnified by climate‐dried forest or severe fire weather, such as low humidity and high air temperatures. Thus, as climate change alters fire regimes, this should be anticipated to impact freshwater ecosystems. Reciprocally, wildfire can influence global climate cycles by increasing terrestrial carbon loss and decreasing carbon storage (Hudiburg et al. 2023). Therefore, climate change will influence fires, and fires will influence climate change.

Climate change and wildfires have many different impacts that will combine for a variety of compounding and cumulative effects on freshwater ecosystems. If climate change and wildfire have shared symptoms, then wildfire and climate change can combine to push stressors or conditions to harmful levels (Moore et al. 2025). With regards to water temperatures, both wildfire and climate change warm waters that control habitat suitability for exothermic species. For example, climate change is incrementally warming streams within western North American watersheds, with a long‐term average increase of 0.27°C/decade, but water temperature in stream regions burned by large wildfires increased 2–3 times faster, which contracts suitable habitat for thermally‐sensitive fishes (Isaak et al. 2010; Falke et al. 2015). Contrastingly, other studies have found population persistence of salmonids despite a severe wildfire driving increases of stream warming of 6°C–7°C in a cool, shaded headwaters stream (Warren et al. 2022). However, long‐term or sublethal impacts beyond the 2‐month study period were not assessed, and complex interactions between fire and landscape characteristics could result in varying responses in habitat and local fish populations (Warren et al. 2022). Alternatively, wildfire smoke can reduce solar radiation and has been found to provide short‐term cooling to summer stream temperatures (David et al. 2018). Other symptoms of climate change and wildfire may interact in more complicated ways. For example, climate change is decreasing snowpack, altering local precipitation, and reducing summer baseflows in some regions, but more extreme precipitation events related to climate change may also increase the frequency, magnitude, and flashiness of storm flows. In the years immediately following high severity wildfires, runoff and the likelihood and magnitude of flooding can also increase at the watershed scale; however, in some cases, low flows have also been observed to increase after wildfires due to the decrease in evapotranspiration (e.g., Niemeyer et al. 2020). Thus, climate change and wildfire could, at least at short timescales, either offset or exacerbate each other's impacts, but both represent stressors that can push key environmental conditions to levels that may start to cause severe ecological harm.

For the majority of regions worldwide where post‐fire debris flows have previously been documented, projected increases in both fire activity and precipitation are anticipated to result in associated increases in post‐fire natural hazard occurrence (McGuire et al. 2024). Flooding, debris flows, and landslides—all of which can occur following moderate‐to‐high severity wildfires in steeper terrain—are capable of both degrading and rejuvenating freshwater habitats and ecosystems. Which of these dichotomous outcomes occurs, though, may depend at least in part on the spatiotemporal scale over which the effects are considered. For example, the effects of post‐fire hydrogeomorphic disturbances may initially appear detrimental to ecosystems at a reach‐ or tributary‐scale, such as channels where a post‐fire debris flow is actually generated and/or transported. In such locations, a stream might experience localized scouring of the streambed, reductions in hydraulic and habitat complexity, such as provided by large woody debris, or even the extirpation of subpopulations of fish (Roghair et al. 2002; Sedell et al. 2015; Curtis et al. 2025). However, in downstream reaches where debris flow material is deposited, the same disturbance may result in substantial influxes of new boulders, sand, and gravel, and/or large wood. These deposits have localized, short‐term effects on stream habitat and can erode by the end of the next year's freshet depending on the local hydrogeomorphic conditions (Ridgway et al. 2024). Over time, these materials will be transported downstream and redistributed throughout the river system (Murphy et al. 2019), diffusing most localized erosion or sedimentation impacts. Thus, the net effect at the watershed‐scale should be an augmentation of sediment to rivers (e.g., spawning gravels) as well as the introduction of new large wood and boulders that enhance habitat complexity. Moreover, in well‐connected river systems with minimal barriers to movement, fish will be able to eventually recolonize any extirpated tributaries (Jager et al. 2021). Post‐disturbance recolonization can result in initial rebounds to the subpopulation abundance that exceed pre‐disturbance levels (Gresswell 1999; Roghair et al. 2002), and given limited fragmentation, buffer any major long‐term impacts to the metapopulation (Neville et al. 2009). In more fragmented watersheds, where hydrological and sediment connectivity are limited and physical barriers restrict recolonization, wildfires and associated post‐fire disturbances pose greater risks to structurally isolated freshwater subpopulations (Sedell et al. 2015). Although wildfires and landslides are natural disturbances that have historically shaped mountainous forested landscapes (Kirchner et al. 2001), increasing river fragmentation (Spinti et al. 2023), combined with climate change‐driven increases in fire frequency, severity, and synchronicity (McGuire et al. 2024), may amplify their impacts on freshwater ecosystems.

There are thus major knowledge gaps in how climate change will interact with wildfire impacts. The impacts of climate change and wildfire on freshwater ecosystems can be considered through a lens of cumulative effects (Schindler 2001; Schindler and Smol 2006). Both pressures have a myriad of different pathways of effects that play out across scales of time and space. In addition, impacts could be additive, synergistic, or antagonistic. The impacts of these different effects and their potential interactions will also likely be context‐dependent, depending on local environmental conditions, geographic features, and other human pressures and management systems. There is also increasing appreciation that climate change is leading to extreme climate events in freshwater ecosystems, such as heat waves and droughts with profound consequences for aquatic biodiversity (Tonkin et al. 2026). These extreme climate events could co‐occur with wildfire impacts by chance or increase the chance of a severe wildfire and thus lead to compounding effects. Thus, wildlife and its impacts will play out over a complicated mosaic of climate impacts. Climate change will continue to contribute to non‐stationarity in ecosystems, and past relationships between wildlife and aquatic ecosystems will shift.

## 4 Opportunities for Advancing Research and Practice

Addressing the growing threat of wildfires to freshwater ecosystems will require shifts in both research design and ultimately management practice. As fire regimes intensify (Rogers et al. 2020; Kirchmeier‐Young et al. 2020), monitoring systems that can capture both acute (e.g., decreases in DO and pH; Curtis et al. 2025, nutrient loading; Silins et al. 2014) and cumulative impacts (e.g., delayed succession changes in freshwater communities; Pugh et al. 2022) are becoming increasingly necessary. This includes the implementation of long‐term monitoring programs across broad spatial and temporal scales (e.g., Biodiversity Observational Networks; Gonzalez et al. 2023) to capture the full trajectory of ecological responses, including immediate, delayed, and cumulative effects. Designing monitoring networks that combine ecological surveys, hydrological monitoring (Ridgway et al. 2024), and emerging tools like remote sensing (Leblon et al. 2012) will enable stronger experimental designs such as BACI studies, allowing researchers to document ecosystem‐level changes, assess wildfire impacts relative to baselines, and monitor for time‐lagged effects like post‐fire natural hazards (Graber et al. 2023) and successional changes in freshwater communities (Dorado‐Guerrero and Willemen 2022).

Consistency in methods and standardization of reporting represents another key opportunity. This includes standardizing definitions and benchmarks across studies, such as volumetric discharge, nutrient/PTE concentrations, water temperature, population density, and community composition, as well as fire severity, intensity, and freshwater biodiversity indicators (Sergio et al. *in review*). This will enable cross‐site comparison and support more robust conclusions across regions (e.g., Nguyen et al. 2025). Such standards could also facilitate meta‐analyses and improve the translation of data into evidence‐based management decisions. Embedded in this is the need to adopt interdisciplinary frameworks to address the limitations stemming from the separate evolution of fire ecology and freshwater science, each with distinct methods, terminology, and management strategies (Schultz et al. 2019). Incorporating insights from both of these fields alongside restoration ecology, hydrology, biogeochemistry, landscape ecology, forest management, geomorphology, and climate science can generate more comprehensive understandings of how wildfires affect freshwater ecosystems.

Empowering Indigenous‐led research (Wong et al. 2025), supporting Indigenous‐led cultural burning, and transforming wildfire policies that uphold Indigenous Knowledge (Hoffman, Christianson, Gray, and Daniels 2022) will enhance freshwater ecosystem management in the face of changing wildfire regimes (e.g., Hoffman et al. 2021). Indigenous Knowledge Holders offer unique (e.g., place‐based) and long‐term knowledge regarding the interactions between wildfires and freshwater ecosystems. Many communities have observed how freshwater systems respond to fire over decades or centuries, including changes associated with increasing fire severity and frequency. For example, cultural burns are grounded in Indigenous ways of knowing that recognize the connections between fire and ecosystem health (Kimmerer and Lake 2001; Eisenberg et al. 2019). While numerous studies have examined the terrestrial outcomes of cultural burning (e.g., Hankins 2013; Eisenberg et al. 2019), much less is known about their effects on freshwater systems (Greenler et al. 2024). When invited and conducted in a “good way” (i.e., meaning research that is relational, culturally grounded, respectful of Indigenous sovereignty, and conducted with free, prior, and informed consent using Indigenous methodologies; see Gordon and Around Him 2024), co‐production of wildfire research and management strategies provides an opportunity to uphold Indigenous Knowledge Systems, thereby addressing both cultural and ecological priorities and revealing insights that would not be captured through Western scientific monitoring alone.

Complementary to cultural burns are low‐severity prescribed burns, which offer additional opportunities for wildfire research and management. Prescribed burning is well documented as vital for biodiversity (Brodie et al. 2024), but these potential benefits are understudied in freshwater ecosystems. Prescribed burning may help to mitigate wildfire risk and support the regeneration of vegetation (i.e., peat‐forming species) in certain peatland types (Marrs et al. 2019) and to restore wetlands where woody encroachment has occurred (Luvuno et al. 2016), but these benefits do not apply to other peatland and wetland types (Baird et al. 2019). An additional consideration when conducting prescribed burns is expanding the scope of effects assessment beyond the focal ecosystems (e.g., downstream or cumulative effects; Hahn et al. 2019; Beyene et al. 2023).

Building on existing agency led fire management practices will be critical for assessing their impacts on freshwater ecosystems and informing strategies to enhance their effectiveness (Rieman et al. 2010). Among the three main phases of fire management, (a) prevention, (b) active fire suppression, and (c) recovery and restoration, there are opportunities to advance the science of fire management in relation to freshwater ecosystems at every stage. In the prevention phase, advancing freshwater‐focused fire management includes understanding aspects such as how chronic fire suppression (Backer et al. 2004), prescribed burns (as previously discussed), or the creation of fuel breaks (e.g., Roche et al. 2024; see Table 1 for glossary) alter hydrological processes, sediment dynamics, and riparian habitat connectivity, ultimately influencing the resilience of freshwater ecosystems to future fires. Next, during the active firefighting phase, there are known impacts associated with strategies such as the application of aerial fire retardants (Dietrich et al. 2013) and direct water withdrawal from streams and lakes (Fletcher et al. 2025). Finally, during the restoration and recovery phase, activities such as salvage logging (Silins et al. 2014) have also been documented to impact freshwater ecosystems. These examples represent only a small subset of fire management practices currently in use within North America, and further research is needed to better understand their implications for freshwater ecosystems.

Effective freshwater‐wildfire research also requires bridging ecological and physical knowledge with decision‐making processes and recognizing the cultural and social dimensions of wildfire management (Bacciu et al. 2022; Moreira et al. 2020). Integrative research approaches that combine ecological monitoring with qualitative data, such as interviews with various stakeholders (e.g., Nocentini et al. 2017; Schultz et al. 2021) and rightsholders, and collaboration with land managers, fire personnel, and community members, can reveal how freshwater ecosystems are, or are not, being accounted for in wildfire management decisions (Schultz et al. 2019). There is an opportunity within wildfire research to apply knowledge‐action frameworks, such as those proposed by Nguyen et al. (2017) to turn knowledge into effective conservation strategies and natural resource management as more empirical evidence about wildfire effects is gathered. When such frameworks are used, alongside the pairing of qualitative and ecological data, there is a greater opportunity to inform more adaptive, inclusive wildfire management strategies, ultimately fostering resilient freshwater ecosystems while addressing cumulative and long‐term wildfire impacts.

## 5 Conclusion

Fire is not inherently detrimental. In many landscapes, it serves as a fundamental ecological process that shapes rivers, wetlands, and riparian forests by renewing habitat structure, redistributing wood and sediment, cycling nutrients, and sustaining biodiversity. The primary threat to freshwater ecosystems today arises not from fire itself but from the transformation of fire regimes. Climate warming, extended drought, fuel accumulation following decades of suppression, the loss of cultural burning, land‐use change, and hydrological alteration have produced fires that are more frequent, extensive, and severe than those to which many ecosystems are adapted. Despite this growing recognition, empirical evidence linking these altered fire regimes to freshwater ecosystem responses remains limited and fragmented, constraining our ability to generalize outcomes or predict impacts across systems. This gap is not simply one of synthesis, but reflects a broader lack of mechanistic, watershed‐scale, and comparative studies needed to understand how changing fire regimes translate into ecological responses in freshwater environments.

Here, we identified seven key research gaps concerning the interactions between fire and freshwater ecosystems. Specifically, these gaps encompass (1) species‐ and community‐level responses, (2) changes to physical and habitat structure, (3) alterations in physicochemical conditions, (4) fire‐informed restoration approaches, (5) building ecosystem resilience, (6) interactions with climate change, and (7) cumulative and cross‐scale impacts. We also highlighted opportunities to advance research and practice, including the need for greater consistency in methods and standardized reporting, empowering and uplifting Indigenous‐led research and supporting cultural burns, learning from prescribed burns, scientifically evaluating how fire management practices affect freshwater ecosystems, and expanding the inclusion of social science perspectives. We hope that this synthesis will further highlight the knowledge gaps regarding interactions between wildfires and freshwater ecosystems, helping to establish clear research priorities and ensuring that these fragile systems are meaningfully considered in future management and policy decisions.

## Author Contributions

**Colin P. R. McCarter:** writing – original draft, writing – review and editing. **Prabha A. Rupasinghe:** writing – original draft, writing – review and editing. **Caliyena R. Brown:** writing – original draft, visualization, writing – review and editing. **Jonathan W. Moore:** writing – original draft, writing – review and editing. **Brendan P. Murphy:** writing – original draft, writing – review and editing. **Rebecca L. Flitcroft:** writing – original draft, writing – review and editing. **Waverley S. Birch:** writing – original draft, writing – review and editing. **Brooke E. Penaluna:** writing – original draft, writing – review and editing. **Victoria Steblaj:** writing – original draft, writing – review and editing. **Meg A. Krawchuk:** writing – original draft, writing – review and editing. **Morgan L. Piczak:** conceptualization, investigation, writing – original draft, writing – review and editing, supervision, project administration. **Ava J. A. Sergio:** writing – original draft, writing – review and editing. **Chantel E. Markle:** writing – original draft, writing – review and editing, project administration, supervision, conceptualization. **Sophie L. Wilkinson:** writing – original draft, writing – review and editing.

## Conflicts of Interest

The authors declare no conflicts of interest.

## Data Availability

Data sharing not applicable to this article as no datasets were generated or analysed during the current study.

## References

[gcb70945-bib-0001] Abatzoglou, J. T. , C. A. Kolden , A. P. Williams , J. A. Lutz , and A. M. S. Smith . 2017. “Climatic Influences on Interannual Variability in Regional Burn Severity Across Western US Forests.” International Journal of Wildland Fire 26, no. 4: 269–275. 10.1071/WF16165.

[gcb70945-bib-0002] Abraham, J. , K. Dowling , and S. Florentine . 2017. “Risk of Post‐Fire Metal Mobilization Into Surface Water Resources: A Review.” Science of the Total Environment 599‐600: 1740–1755. 10.1016/j.scitotenv.2017.05.096. 28535601

[gcb70945-bib-0003] Ackley, C. , S. E. Tank , K. M. Haynes , F. Rezanezhad , C. McCarter , and W. L. Quinton . 2021. “Coupled Hydrological and Geochemical Impacts of Wildfire in Peatland‐Dominated Regions of Discontinuous Permafrost.” Science of the Total Environment 782: 146841. 10.1016/j.scitotenv.2021.146841. 33848861

[gcb70945-bib-0004] Agee, J. K. , and C. N. Skinner . 2005. “Basic Principles of Forest Fuel Reduction Treatments.” Forest Ecology and Management 211: 83–96.

[gcb70945-bib-0315] Aldous, A. , J. Fitzsimons , B. Richter , and L. Bach . 2011. “Droughts, Floods and Freshwater Ecosystems: Evaluating Climate Change Impacts and Developing Adaptation Strategies.” Marine and Freshwater Research 62, no. 3: 223–231.

[gcb70945-bib-0005] Alexander, M. E. , and D. Quintilio . 1990. “Perspectives on Experimental Fires in Canadian Forestry Research.” Mathematical and Computer Modelling 13, no. 12: 17–26.

[gcb70945-bib-0006] Aliaga‐Rossel, E. , D. Edinger , M. Marmontel , L. Guizada Duran , and A. Fahlman . 2025. “Forest Fire Smoke as a Threat to the Health of River Dolphins.” Conservation Biology 39: e70098. 10.1111/cobi.70098. 40551632 PMC12658923

[gcb70945-bib-0007] Allingham, S. M. , S. J. Drake , A. Ramsey , C. D. Field , F. C. Nwaishi , and D. R. Elliott . 2024. “Changes in Nitrogen Functional Genes and Microbial Populations in Soil Profiles of a Peatland Under Different Burning Regimes.” Applied Soil Ecology 200: 105426. 10.1016/j.apsoil.2024.105426.

[gcb70945-bib-0008] Anderson, N. J. 2014. “Landscape Disturbance and Lake Response: Temporal and Spatial Perspectives.” Freshwater Reviews 7, no. 2: 77–120.

[gcb70945-bib-0009] Arkle, R. S. , and D. S. Pilliod . 2010. “Prescribed Fires as Ecological Surrogates for Wildfires: A Stream and Riparian Perspective.” Forest Ecology and Management 259, no. 5: 893–903.

[gcb70945-bib-0010] Arkle, R. S. , D. S. Pilliod , and J. L. Welty . 2012. “Pattern and Process of Prescribed Fires Influence Effectiveness at Reducing Wildfire Severity in Dry Coniferous Forests.” Forest Ecology and Management 276: 174–184.

[gcb70945-bib-0331] Arroyo‐Morales, R. , R. Reques , R. Real , and D. Romero . 2023. “Extreme Weather Event Disrupts Reproduction of an Isolated Western Spadefoot Toad Population, *Pelobates cultripes* (Cuvier, 1829), at its Southern Range Limit.” Animal Biodiversity and Conservation 46, no. 1: 47–52.

[gcb70945-bib-0011] Bacciu, V. , C. Sirca , and D. Spano . 2022. “Towards a Systemic Approach to Fire Risk Management.” Environmental Science & Policy 129: 37–44.

[gcb70945-bib-0012] Backer, D. M. , S. E. Jensen , and G. R. McPherson . 2004. “Impacts of Fire‐Suppression Activities on Natural Communities.” Conservation Biology 18, no. 4: 937–946.

[gcb70945-bib-0013] Bailey, L. L. , R. Henderson , W. A. Estes‐Zumpf , et al. 2025. “Unburned Habitat Essential for Amphibian Breeding Persistence Following Wildfire.” Global Ecology and Conservation 57: e03389. 10.1016/j.gecco.2024.e03389.

[gcb70945-bib-0316] Baird, A. J. , C. D. Evans , R. Mills , et al. 2019. “Validity of Managing Peatlands With Fire.” Nature Geoscience 12, no. 11: 884–885.

[gcb70945-bib-0014] Bakhshaii, A. , and E. A. Johnson . 2019. “A Review of a New Generation of Wildfire–Atmosphere Modeling.” Canadian Journal of Forest Research 49, no. 6: 565–574.

[gcb70945-bib-0015] Baron, J. N. , S. E. Gergel , P. F. Hessburg , and L. D. Daniels . 2022. “A Century of Transformation: Fire Regime Transitions From 1919 to 2019 in Southeastern British Columbia, Canada.” Landscape Ecology 37, no. 10: 2707–2727.

[gcb70945-bib-0016] BCWS; British Columbia Wildfire Service . 2024. Fuel Management Practices Guide. https://www2.gov.bc.ca/assets/gov/public‐safety‐and‐emergency‐services/wildfire‐status/prevention/fire‐fuel‐management/fuels‐management/2024_fuelmanagementpracticesguide1.pdf.

[gcb70945-bib-0017] Beakes, M. P. , J. W. Moore , S. A. Hayes , and S. M. Sogard . 2014. “Wildfire and the Effects of Shifting Stream Temperature on Salmonids.” Ecosphere 5, no. 5: 1–14.

[gcb70945-bib-0018] Beechie, T. J. , D. A. Sear , J. D. Olden , et al. 2010. “Process‐Based Principles for Restoring River Ecosystems.” Bioscience 60, no. 3: 209–222.

[gcb70945-bib-0019] Benali, A. , B. Mota , N. Carvalhais , et al. 2017. “Bimodal fire regimes unveil a global‐scale anthropogenic fingerprint.” Global Ecology and Biogeography 26, no. 7: 799–811.

[gcb70945-bib-0021] Benscoter, B. W. , D. K. Thompson , J. M. Waddington , et al. 2011. “Interactive Effects of Vegetation, Soil Moisture and Bulk Density on Depth of Burning of Thick Organic Soils.” International Journal of Wildland Fire 20, no. 3: 418–429.

[gcb70945-bib-0022] Beyene, M. T. , S. G. Leibowitz , C. J. Dunn , and K. D. Bladon . 2023. “To Burn or Not to Burn: An Empirical Assessment of the Impacts of Wildfires and Prescribed Fires on Trace Element Concentrations in Western US Streams.” Science of the Total Environment 863: 160731. 36502971 10.1016/j.scitotenv.2022.160731 PMC9988007

[gcb70945-bib-0023] Bisson, P. A. , B. E. Rieman , C. Luce , et al. 2003. “Fire and Aquatic Ecosystems of the Western USA: Current Knowledge and Key Questions.” Forest Ecology and Management 178, no. 1–2: 213–229.

[gcb70945-bib-0024] Bixby, R. J. , S. D. Cooper , R. E. Gresswell , L. E. Brown , C. N. Dahm , and K. A. Dwire . 2015. “Fire Effects on Aquatic Ecosystems: An Assessment of the Current State of the Science.” Freshwater Science 34, no. 4: 1340–1350.

[gcb70945-bib-0025] Bladon, K. D. , M. B. Emelko , U. Silins , and M. Stone . 2014. “Wildfire and the Future of Water Supply.” Environmental Science & Technology 48, no. 16: 8936–8943. 25007310 10.1021/es500130g

[gcb70945-bib-0028] Bowen, L. , A. K. Miles , C. A. Kolden , et al. 2015. “Effects of Wildfire on Sea Otter ( *Enhydra lutris* ) Gene Transcript Profiles.” Marine Mammal Science 31, no. 1: 191–210. 10.1111/mms.12151.

[gcb70945-bib-0029] Bowman, D. M. , J. Balch , P. Artaxo , et al. 2011. “The Human Dimension of Fire Regimes on Earth.” Journal of Biogeography 38, no. 12: 2223–2236. 22279247 10.1111/j.1365-2699.2011.02595.x PMC3263421

[gcb70945-bib-0030] Bowman, D. M. , C. A. Kolden , J. T. Abatzoglou , F. H. Johnston , G. R. van der Werf , and M. Flannigan . 2020. “Vegetation Fires in the Anthropocene.” Nature Reviews Earth & Environment 1, no. 10: 500–515.

[gcb70945-bib-0317] Brodie, E. G. , E. E. Knapp , W. R. Brooks , S. A. Drury , and M. W. Ritchie . 2024. “Forest Thinning and Prescribed Burning Treatments Reduce Wildfire Severity and Buffer the Impacts of Severe Fire Weather.” Fire Ecology 20, no. 1: 17.

[gcb70945-bib-0032] Brown, L. E. , J. Holden , S. M. Palmer , K. Johnston , S. J. Ramchunder , and R. Grayson . 2015. “Effects of Fire on the Hydrology, Biogeochemistry, and Ecology of Peatland River Systems.” Freshwater Science 34, no. 4: 1406–1425.

[gcb70945-bib-0033] Buma, B. , K. Hayes , S. Weiss , and M. Lucash . 2022. “Short‐Interval Fires Increasing in the Alaskan Boreal Forest as Fire Self‐Regulation Decays Across Forest Types.” Scientific Reports 12, no. 1: 4901. 10.1038/s41598-022-08912-8. 35318377 PMC8941092

[gcb70945-bib-0035] Burton, T. A. 2005. “Fish and Stream Habitat Risks From Uncharacteristic Wildfire: Observations From 17 Years of Fire‐Related Disturbances on the Boise National Forest, Idaho.” Forest Ecology and Management 211, no. 1–2: 140–149.

[gcb70945-bib-0036] Bury, R. B. , D. J. Major , and D. Pilliod . 2002. “Responses of Amphibians to Fire Disturbance in Pacific Northwest Forests: A Review.” In Proceedings: The Role of Fire for Nongame Wildlife Management and Community Restoration: Traditional Uses and New Directions. Gen. Tech. Rep. NE‐288, edited by W. M. Ford , K. R. Russell , and C. E. Moorman , 34–42. US Dept. of Agriculture, Forest Service, Northeastern Research Station.

[gcb70945-bib-0037] Cafferata, P. H. , D. B. Coe , and W. R. Short . 2021. “Sixty Years of Post‐Fire Assessment and Monitoring on Non‐Federal Lands in California: What Have We Learned?” Environmental & Engineering Geoscience 27, no. 4: 409–422.

[gcb70945-bib-0038] Caldwell, C. A. , G. Z. Jacobi , M. C. Anderson , et al. 2013. “Prescribed‐Fired Effects on an Aquatic Community of a Southwest Montane Grassland System.” North American Journal of Fisheries Management 33: 1049–1062.

[gcb70945-bib-0039] Caltabellotta, G. , M. Iovino , and V. Bagarello . 2022. “Intensity and Persistence of Water Repellency at Different Soil Moisture Contents and Depths After a Forest Wildfire.” Journal Of Hydrology And Hydromechanics 70, no. 4: 410–420.

[gcb70945-bib-0040] Campos, I. , and N. Abrantes . 2021. “Forest Fires as Drivers of Contamination of Polycyclic Aromatic Hydrocarbons to the Terrestrial and Aquatic Ecosystems.” Current Opinion in Environmental Science & Health 24: 100293.

[gcb70945-bib-0041] Campos, I. , C. Vale , N. Abrantes , J. J. Keizer , and P. Pereira . 2015. “Effects of Wildfire on Mercury Mobilisation in Eucalypt and Pine Forests.” Catena 131: 149–159.

[gcb70945-bib-0318] Cannon, S. H. , E. M. Boldt , J. L. Laber , J. W. Kean , and D. M. Staley . 2011. “Rainfall Intensity–Duration Thresholds for Postfire Debris‐Flow Emergency‐Response Planning.” Natural Hazards 59, no. 1: 209–236.

[gcb70945-bib-0319] Carpenter, S. R. , O. Kinne , and W. Wieser . 2003. Regime Shifts in Lake Ecosystems: Pattern and Variation. Vol. 15. International Ecology Institute.

[gcb70945-bib-0043] Carvalho, F. , A. Pradhan , N. Abrantes , et al. 2019. “Wildfire Impacts on Freshwater Detrital Food Webs Depend on Runoff Load, Exposure Time and Burnt Forest Type.” Science of the Total Environment 692: 691–700. 31539977 10.1016/j.scitotenv.2019.07.265

[gcb70945-bib-0044] Casey, R. E. , and S. J. Klaine . 2001. “Nutrient Attenuation by a Riparian Wetland During Natural and Artificial Runoff Events.” Journal of Environmental Quality 30, no. 5: 1720–1731. 11577881 10.2134/jeq2001.3051720x

[gcb70945-bib-0045] Certini, G. 2005. “Effects of Fire on Properties of Forest Soils: A Review.” Oecologia 143: 1–10. 15688212 10.1007/s00442-004-1788-8

[gcb70945-bib-0046] Chanasyk, D. S. , I. R. Whitson , E. Mapfumo , J. M. Burke , and E. E. Prepas . 2003. “The Impacts of Forest Harvest and Wildfire on Soils and Hydrology in Temperate Forests: A Baseline to Develop Hypotheses for the Boreal Plain.” Journal of Environmental Engineering and Science 2, no. S1: S51–S62.

[gcb70945-bib-0047] Charette, T. , and E. E. Prepas . 2003. “Wildfire Impacts on Phytoplankton Communities of Three Small Lakes on the Boreal Plain, Alberta, Canada: A Paleolimnological Study.” Canadian Journal of Fisheries and Aquatic Sciences 60, no. 5: 584–593.

[gcb70945-bib-0048] Chasmer, L. , C. Mahoney , K. Millard , et al. 2020. “Remote Sensing of Boreal Wetlands 2: Methods for Evaluating Boreal Wetland Ecosystem State and Drivers of Change.” Remote Sensing 12, no. 8: 1321.

[gcb70945-bib-0051] Coble, A. A. , B. E. Penaluna , L. J. Six , and J. Verschuyl . 2023. “Fire Severity Influences Large Wood and Stream Ecosystem Responses in Western Oregon Watersheds.” Fire Ecology 19: 1–21.

[gcb70945-bib-0052] Connell, J. H. , and W. P. Sousa . 1983. “On the Evidence Needed to Judge Ecological Stability or Persistence.” American Naturalist 121, no. 6: 789–824.

[gcb70945-bib-0320] Cooper, S. D. , K. Klose , D. B. Herbst , J. White , S. M. Drenner , and E. J. Eliason . 2021. “Wildfire and Drying Legacies and Stream Invertebrate Assemblages.” Freshwater Science 40, no. 4: 659–680.

[gcb70945-bib-0053] Cooper, S. D. , H. M. Page , S. W. Wiseman , et al. 2015. “Physicochemical and Biological Responses of Streams to Wildfire Severity in Riparian Zones.” Freshwater Biology 60, no. 12: 2600–2619.

[gcb70945-bib-0054] Cramp, R. , C. Mulvey , J. Cameron , M. Wintour , D. Gomez Isaza , and C. Franklin . 2021. Impacts of Post‐Fire Ash and Runoff Sediment on the Physiological Tolerances of Australian Freshwater Aquatic fauna. NESP Threatened Species Recovery Hub Project 8.3.7 Report, Brisbane.

[gcb70945-bib-0055] Crawford, A. J. , C. M. Belcher , S. New , et al. 2024. “Tropical Peat Composition May Provide a Negative Feedback on Fire Occurrence and Severity.” Nature Communications 15, no. 1: 7363. 10.1038/s41467-024-50916-7 PMC11349947 39191729

[gcb70945-bib-0056] Curtis, J. A. , G. S. Johnson , J. D. Cahill , et al. 2025. “2022 McKinney Rain‐On‐Wildfire Event, Dissolved Oxygen Sags, and a Fish Kill on the Klamath River, California.” Scientific Reports 15, no. 1: 24668. 40634423 10.1038/s41598-025-08179-9 PMC12241632

[gcb70945-bib-0057] Dang, A. T. , L. Kumar , M. Reid , and O. Mutanga . 2021. “Fire Danger Assessment Using Geospatial Modelling in Mekong Delta, Vietnam: Effects on Wetland Resources.” Remote Sensing Applications: Society and Environment 21: 100456.

[gcb70945-bib-0058] Daniels, L. D. , S. Dickson‐Hoyle , J. N. Baron , et al. 2024. “The 2023 Wildfires in British Columbia, Canada: Impacts, Drivers, and Transformations to Coexist With Wildfire.” Canadian Journal of Forest Research 55: 1–18.

[gcb70945-bib-0059] David, A. T. , J. E. Asarian , and F. K. Lake . 2018. “Wildfire Smoke Cools Summer River and Stream Water Temperatures.” Water Resources Research 54, no. 10: 7273–7290.

[gcb70945-bib-0061] DeBano, L. F. 1981. Water Repellent Soils: A State‐Of‐The‐Art. Vol. 46. US Department of Agriculture, Forest Service, Pacific Southwest Forest and Range Experiment Station.

[gcb70945-bib-0062] Delay, S. J. , O. Urquhart , and J. D. Litzgus . 2024. “Wind Farm and Wildfire: Spatial Ecology of an Endangered Freshwater Turtle in a Recovering Landscape.” Canadian Journal of Zoology 102, no. 2: 124–146.

[gcb70945-bib-0063] Dietrich, J. P. , M. S. Myers , S. A. Strickland , A. Van Gaest , and M. R. Arkoosh . 2013. “Toxicity of Forest Fire Retardant Chemicals to Stream‐Type Chinook Salmon Undergoing Parr–Smolt Transformation.” Environmental Toxicology and Chemistry 32, no. 1: 236–247. 23161484 10.1002/etc.2052

[gcb70945-bib-0064] Doerr, S. H. , R. A. Shakesby , and R. Walsh . 2000. “Soil Water Repellency: Its Causes, Characteristics and Hydro‐Geomorphological Significance.” Earth‐Science Reviews 51, no. 1–4: 33–65.

[gcb70945-bib-0065] Dorado‐Guerrero, B. , and L. Willemen . 2022. “Assessing the Impact of Post‐Fire Restoration Interventions Using Spectral Vegetation Indices.” In 13th European Conference on Ecological Restoration, SER Europe 2022.

[gcb70945-bib-0066] Dudgeon, D. , A. H. Arthington , M. O. Gessner , et al. 2006. “Freshwater Biodiversity: Importance, Threats, Status and Conservation Challenges.” Biological Reviews 81, no. 2: 163–182. 16336747 10.1017/S1464793105006950

[gcb70945-bib-0067] Dunham, J. B. , A. E. Rosenberger , C. H. Luce , and B. E. Rieman . 2007. “Influences of Wildfire and Channel Reorganization on Spatial and Temporal Variation in Stream Temperature and the Distribution of Fish and Amphibians.” Ecosystems 10, no. 2: 335–346.

[gcb70945-bib-0502] Dunham, J. B. , M. K. Young , R. E. Gresswell , and B. E. Rieman . 2003. “Effects of Fire on Fish Populations: Landscape Perspectives on Persistence of Native Fishes and Nonnative Fish Invasions.” Forest Ecology and Management 178, no. 1‐2: 183–196.

[gcb70945-bib-0068] Dwire, K. A. , K. E. Meyer , G. Riegel , and T. Burton . 2016. “Riparian Fuel Treatments in the Western USA: Challenges and Considerations.” Joint Fire Science Program Synthesis Reports. 30. https://digitalcommons.unl.edu/jfspsynthesis/30.

[gcb70945-bib-0069] Earl, N. O. , J. J. M. de Klein , and A. S. Mehring . 2025. “Black and White Fire Ash Alters Greenhouse Gas Emissions and Temporarily Reverses Carbon Source‐Sink Status in Aquatic Mesocosms.” Environmental Science & Technology 59: 10990–11001. 40436645 10.1021/acs.est.4c10046

[gcb70945-bib-0070] Earl, S. R. , and D. W. Blinn . 2003. “Effects of Wildfire Ash on Water Chemistry and Biota in South‐Western USA Streams.” Freshwater Biology 48, no. 6: 1015–1030.

[gcb70945-bib-0071] Eaton, B. C. , R. D. Moore , and T. R. Giles . 2010. “Forest Fire, Bank Strength and Channel Instability: The ‘Unusual’ Response of Fishtrap Creek, British Columbia.” Earth Surface Processes and Landforms 35, no. 10: 1167–1183.

[gcb70945-bib-0072] Eisenberg, C. , C. L. Anderson , A. Collingwood , et al. 2019. “Out of the Ashes: Ecological Resilience to Extreme Wildfire, Prescribed Burns, and Indigenous Burning in Ecosystems.” Frontiers in Ecology and Evolution 7: 436.

[gcb70945-bib-0074] Elmes, M. C. , D. K. Thompson , and J. S. Price . 2019. “Changes to the Hydrophysical Properties of Upland and Riparian Soils in a Burned Fen Watershed in the Athabasca Oil Sands Region, Northern Alberta, Canada.” Catena 181: 104077. 10.1016/j.catena.2019.104077.

[gcb70945-bib-0075] Emelko, M. B. , U. Silins , K. D. Bladon , and M. Stone . 2011. “Implications of Land Disturbance on Drinking Water Treatability in a Changing Climate: Demonstrating the Need for “Source Water Supply and Protection” Strategies.” Water Research 45, no. 2: 461–472. 10.1016/j.watres.2010.08.051. 20951401

[gcb70945-bib-0076] Emmerton, C. A. , C. A. Cooke , S. Hustins , et al. 2020. “Severe Western Canadian Wildfire Affects Water Quality Even at Large Basin Scales.” Water Research 183: 116071. 10.1016/j.watres.2020.116071. 32717650

[gcb70945-bib-0077] Enache, M. , and Y. T. Prairie . 2000. “Paleolimnological Reconstruction of Forest Fire Induced Changes in Lake Biogeochemistry (Lac Francis, Abitibi, Quebec, Canada).” Canadian Journal of Fisheries and Aquatic Sciences 57, no. S2: 146–154.

[gcb70945-bib-0078] Erdozain, M. , A. Cardil , and S. de‐Miguel . 2024. “Fire Impacts on the Biology of Stream Ecosystems: A Synthesis of Current Knowledge to Guide Future Research and Integrated Fire Management.” Global Change Biology 30, no. 7: e17389. 38984506 10.1111/gcb.17389

[gcb70945-bib-0079] Fairfax, E. , and A. Whittle . 2020. “Smokey the Beaver: Beaver‐Dammed Riparian Corridors Stay Green During Wildfire Throughout the Western United States.” Ecological Applications 30, no. 8: e02225. 32881199 10.1002/eap.2225

[gcb70945-bib-0081] Falke, J. A. , R. L. Flitcroft , J. B. Dunham , K. M. McNyset , P. F. Hessburg , and G. H. Reeves . 2015. “Climate Change and Vulnerability of Bull Trout ( *Salvelinus confluentus* ) in a Fire‐Prone Landscape.” Canadian Journal of Fisheries and Aquatic Sciences 72, no. 2: 304–318.

[gcb70945-bib-0082] Farruggia, M. J. , J. Brahney , A. J. Tanentzap , et al. 2024. “Wildfire Smoke Impacts Lake Ecosystems.” Global Change Biology 30, no. 6: e17367. 38840430 10.1111/gcb.17367

[gcb70945-bib-0085] Flanagan, N. E. , H. Wang , S. Winton , and C. J. Richardson . 2020. “Low‐Severity Fire as a Mechanism of Organic Matter Protection in Global Peatlands: Thermal Alteration Slows Decomposition.” Global Change Biology 26, no. 7: 3930–3946. 32388914 10.1111/gcb.15102

[gcb70945-bib-0086] Fletcher, N. , K. Hartwig , and Z. Botelho . 2025. Opinion: B.C. Giving Away Fresh Water While Funding to Fight Fires, Floods and Scarcity Dries Up. Vancouver Sun. https://www.vancouversun.com/opinion/op‐ed/opinion‐bc‐giving‐away‐fresh‐water‐funding‐fires‐floods‐scarcity.

[gcb70945-bib-0088] Furukawa, A. K. , O. F. Sutton , K. L. Simone , et al. 2025. “Hydrological Feedbacks in Northern Peatlands 2: Peat Depth as a Control on Peatland Resilience.” Ecohydrology 18: e70158.

[gcb70945-bib-0089] Gage, H. J. M. , P. A. Moore , B. Mackinnon , G. Granath , S. L. Wilkinson , and J. M. Waddington . 2024. “Assessing Moss Transplant Methods to Enhance Sphagnum Moss Recovery in Post‐Wildfire Hydrophobic Peat.” Ecological Engineering 205: 107292.

[gcb70945-bib-0091] Garcia, L. C. , J. K. Szabo , F. de Oliveira Roque , et al. 2021. “Record‐Breaking Wildfires in the World's Largest Continuous Tropical Wetland: Integrative Fire Management Is Urgently Needed for Both Biodiversity and Humans.” Journal of Environmental Management 293: 112870. 10.1016/j.jenvman.2021.112870. 34052615

[gcb70945-bib-0092] Geary, W. L. , T. S. Doherty , D. G. Nimmo , A. I. Tulloch , and E. G. Ritchie . 2020. “Predator Responses to Fire: A Global Systematic Review and Meta‐Analysis.” Journal of Animal Ecology 89, no. 4: 955–971. 31774550 10.1111/1365-2656.13153

[gcb70945-bib-0093] Gomez Isaza, D. F. , R. L. Cramp , and C. E. Franklin . 2022. “Fire and Rain: A Systematic Review of the Impacts of Wildfire and Associated Runoff on Aquatic Fauna.” Global Change Biology 28, no. 8: 2578–2595. 35038772 10.1111/gcb.16088

[gcb70945-bib-0094] Gonino, G. , P. Branco , E. Benedito , M. T. Ferreira , and J. M. Santos . 2019. “Short‐Term Effects of Wildfire Ash Exposure on Behaviour and Hepatosomatic Condition of a Potamodromous Cyprinid Fish, the Iberian Barbel Luciobarbus Bocagei (Steindachner, 1864).” Science of the Total Environment 665: 226–234. 30772552 10.1016/j.scitotenv.2019.02.108

[gcb70945-bib-0095] Gonzalez, A. , P. Vihervaara , P. Balvanera , et al. 2023. “A Global Biodiversity Observing System to Unite Monitoring and Guide Action.” Nature Ecology & Evolution 7, no. 12: 1947–1952. 37620553 10.1038/s41559-023-02171-0

[gcb70945-bib-0096] González‐Sargas, E. , S. R. Lee , L. G. Perry , and P. B. Shafroth . 2025. “Legacies of a Large Flood and Biological Control on Riparian Vegetation Successional Trajectories Along a Dryland Braided River.” River Research and Applications 41, no. 5: 1169–1185.

[gcb70945-bib-0098] Gordon, H. S. J. , and D. Around Him . 2024. “Conducting Research “in a Good Way”: Relationships as the Foundation of Research.” Arctic Science 11: 1–16.

[gcb70945-bib-0099] Gould, J. , J. Clulow , F. Herb , and S. Clulow . 2022. “An Ephemerality Paradox: Evidence of Virtual Semelparity in Ephemeral Pool‐Breeding Anurans.” Austral Ecology 47, no. 8: 1591–1608.

[gcb70945-bib-0100] Graber, A. P. , M. A. Thomas , and J. W. Kean . 2023. “How Long Do Runoff‐Generated Debris‐Flow Hazards Persist After Wildfire?” Geophysical Research Letters 50, no. 19: e2023GL105101.

[gcb70945-bib-0102] Grantham, T. E. , J. H. Matthews , and B. P. Bledsoe . 2019. “Shifting Currents: Managing Freshwater Systems for Ecological Resilience in a Changing Climate.” Water Security 8: 100049.

[gcb70945-bib-0322] Greenler, S. M. , F. K. Lake , W. Tripp , et al. 2024. “Blending Indigenous and Western Science: Quantifying Cultural Burning Impacts in Karuk Aboriginal Territory.” Ecological Applications 34, no. 4: e2973. 38616644 10.1002/eap.2973

[gcb70945-bib-0103] Grenz, J. 2025. Medicine Wheel for the Planet. Vintage Canada, Penguin Randomhouse Canada.

[gcb70945-bib-0104] Gresswell, R. E. 1999. “Fire and Aquatic Ecosystems in Forested Biomes of North America.” Transactions of the American Fisheries Society 128, no. 2: 193–221.

[gcb70945-bib-0105] Gschwentner, D. , L. Blehm , J. Brahney , D. Wedin , and J. R. Corman . 2025. “Nitrogen‐Limitation Overrides Impacts of Wildfire Ash on Primary Production in Naturally Eutrophic, Grassland Lakes.” Ecosystems 28, no. 6: 63.

[gcb70945-bib-0106] Guêné‐Nanchen, M. , M. C. LeBlanc , and L. Rochefort . 2022. “Post‐Fire Peatland Vegetation Recovery: A Case Study in Open Rich Fens of the Canadian Boreal Forest.” Botany 100, no. 5: 435–447.

[gcb70945-bib-0107] Guo, M. , J. Li , C. Sheng , J. Xu , and L. Wu . 2017. “A Review of Wetland Remote Sensing.” Sensors 17, no. 4: 777. 28379174 10.3390/s17040777 PMC5422050

[gcb70945-bib-0109] Hahn, G. E. , T. A. Coates , R. E. Latham , and H. Majidzadeh . 2019. “Prescribed Fire Effects on Water Quality and Freshwater Ecosystems in Moist‐Temperate Eastern North America.” Natural Areas Journal 39, no. 1: 46–57.

[gcb70945-bib-0111] Hancock, C. A. , and K. Wlodarczyk . 2025. “The Role of Wildfires and Forest Harvesting on Geohazards and Channel Instability During the November 2021 Atmospheric River in Southwestern British Columbia, Canada.” Earth Surface Processes and Landforms 50, no. 1: e6065.

[gcb70945-bib-0112] Hanes, C. C. , X. Wang , P. Jain , M. A. Parisien , J. M. Little , and M. D. Flannigan . 2019. “Fire‐Regime Changes in Canada Over the Last Half Century.” Canadian Journal of Forest Research 49, no. 3: 256–269.

[gcb70945-bib-0113] Hankins, D. L. 2013. “The Effects of Indigenous Prescribed Fire on Riparian Vegetation in Central California.” Ecological Processes 2, no. 1: 24.

[gcb70945-bib-0114] Hardy, C. C. 2005. “Wildland Fire Hazard and Risk: Problems, Definitions, and Context.” Forest Ecology and Management 211, no. 1–2: 73–82.

[gcb70945-bib-0115] Harris, H. E. , C. V. Baxter , and J. M. Davis . 2015. “Debris Flows Amplify Effects of Wildfire on Magnitude and Composition of Tributary Subsidies to Mainstem Habitats.” Freshwater Science 34, no. 4: 1457–1467.

[gcb70945-bib-0116] Harris, M. P. , J. D. Coop , J. A. Balik , J. R. McFarland , S. A. Parks , and C. S. Stevens‐Rumann . 2025. “Aspen Impedes Wildfire Spread in Southwestern United States Landscapes.” Ecological Applications 35, no. 5: e70061. 40621807 10.1002/eap.70061 PMC12231080

[gcb70945-bib-0119] He, T. , B. B. Lamont , and J. G. Pausas . 2019. “Fire as a Key Driver of Earth's Biodiversity.” Biological Reviews 94, no. 6: 1983–2010. 31298472 10.1111/brv.12544

[gcb70945-bib-0120] Hefting, M. , B. Beltman , D. Karssenberg , K. Rebel , M. van Riessen , and M. Spijker . 2006. “Water Quality Dynamics and Hydrology in Nitrate Loaded Riparian Zones in The Netherlands.” Environmental Pollution 139, no. 1: 143–156. 15996804 10.1016/j.envpol.2005.04.023

[gcb70945-bib-0121] Hessburg, P. F. , S. Charnley , A. N. Gray , et al. 2022. “Climate and Wildfire Adaptation of Inland Northwest US Forests.” Frontiers in Ecology and the Environment 20: 40–48.

[gcb70945-bib-0122] Hessburg, P. F. , T. A. Spies , D. A. Perry , et al. 2016. “Tamm Review: Management of Mixed‐Severity Fire Regime Forests in Oregon, Washington, and Northern California.” Forest Ecology and Management 366: 221–250.

[gcb70945-bib-0123] Higuera, P. E. 2019. “First‐ and Second‐Order Fire Effects.” In Encyclopedia of Wildfires and Wildland‐Urban Interface (WUI) Fires, edited by S. Manzello . Springer.

[gcb70945-bib-0124] Hoffman, K. M. , A. C. Christianson , S. Dickson‐Hoyle , et al. 2022. “The Right to Burn: Barriers and Opportunities for Indigenous‐Led Fire Stewardship in Canada.” Facets 7: 464–481. 10.1139/facets-2021-0062.

[gcb70945-bib-0125] Hoffman, K. M. , A. C. Christianson , R. W. Gray , and L. Daniels . 2022. “Western Canada's New Wildfire Reality Needs a New Approach to Fire Management.” Environmental Research Letters 17, no. 6: 061001. 10.1088/1748-9326/ac7345.

[gcb70945-bib-0126] Hoffman, K. M. , E. L. Davis , S. B. Wickham , et al. 2021. “Conservation of Earth's Biodiversity Is Embedded in Indigenous Fire Stewardship.” Proceedings of the National Academy of Sciences of the United States of America 118, no. 32: e2105073118. 10.1073/pnas.2105073118. 34362847 PMC8364180

[gcb70945-bib-0323] Holling, C. S. 1973. “Resilience and Stability of Ecological Systems.” November 1973.

[gcb70945-bib-0127] Hudiburg, T. , J. Mathias , K. Bartowitz , et al. 2023. “Terrestrial Carbon Dynamics in an Era of Increasing Wildfire.” Nature Climate Change 13, no. 12: 1306–1316.

[gcb70945-bib-0128] Isaak, D. J. , C. H. Luce , B. E. Rieman , et al. 2010. “Effects of Climate Change and Wildfire on Stream Temperatures and Salmonid Thermal Habitat in a Mountain River Network.” Ecological Applications 20, no. 5: 1350–1371. 20666254 10.1890/09-0822.1

[gcb70945-bib-0129] Jager, H. I. , J. W. Long , R. L. Malison , et al. 2021. “Resilience of Terrestrial and Aquatic Fauna to Historical and Future Wildfire Regimes in Western North America.” Ecology and Evolution 11, no. 18: 12259–12284. 34594498 10.1002/ece3.8026 PMC8462151

[gcb70945-bib-0130] Jain, P. , S. C. Coogan , S. G. Subramanian , M. Crowley , S. Taylor , and M. D. Flannigan . 2020. “A Review of Machine Learning Applications in Wildfire Science and Management.” Environmental Reviews 28, no. 4: 478–505.

[gcb70945-bib-0131] Jaiswal, D. , U. Pandey , V. Mishra , and J. Pandey . 2021. “Integrating Resilience With Functional Ecosystem Measures: A Novel Paradigm for Management Decisions Under Multiple‐Stressor Interplay in Freshwater Ecosystems.” Global Change Biology 27, no. 16: 3699–3717. 33915017 10.1111/gcb.15662

[gcb70945-bib-0132] Johnston, D. C. , M. R. Turetsky , B. W. Benscoter , and B. M. Wotton . 2015. “Fuel Load, Structure, and Potential Fire Behaviour in Black Spruce Bogs.” Canadian Journal of Forest Research 45, no. 7: 888–899.

[gcb70945-bib-0133] Jones, M. W. , J. T. Abatzoglou , S. Veraverbeke , et al. 2022. “Global and Regional Trends and Drivers of Fire Under Climate Change.” Reviews of Geophysics 60, no. 3: e2020RG000726.

[gcb70945-bib-0134] Karr, J. R. , J. J. Rhodes , G. W. Minshall , et al. 2004. “The Effects of Postfire Salvage Logging on Aquatic Ecosystems in the American West.” Bioscience 54, no. 11: 1029–1033.

[gcb70945-bib-0135] Keeley, J. E. 2009. “Fire Intensity, Fire Severity, and Burn Severity: A Brief Review and Suggested Usage.” International Journal of Wildland Fire 18: 116–126.

[gcb70945-bib-0136] Keeley, J. E. , and J. G. Pausas . 2022. “Evolutionary Ecology of Fire.” Annual Review of Ecology, Evolution, and Systematics 53, no. 1: 203–225.

[gcb70945-bib-0137] Kelly, A. G. , and T. D. Harris . 2024. “Watershed Grassland Fires Drive Nutrient Increases in Replicated Experimental Ponds.” Lake and Reservoir Management 40, no. 3: 303–316.

[gcb70945-bib-0138] Kelly, L. T. , K. M. Giljohann , A. Duane , et al. 2020. “Fire and Biodiversity in the Anthropocene.” Science 370, no. 6519: eabb0355. 33214246 10.1126/science.abb0355

[gcb70945-bib-0139] Kettridge, N. , R. E. Humphrey , J. E. Smith , et al. 2014. “Burned and Unburned Peat Water Repellency: Implications for Peatland Evaporation Following Wildfire.” Journal of Hydrology 513: 335–341.

[gcb70945-bib-0140] Kimmerer, R. W. , and F. K. Lake . 2001. “The Role of Indigenous Burning in Land Management.” Journal of Forestry 99, no. 11: 36–41.

[gcb70945-bib-0141] Kirchmeier‐Young, M. C. , E. Malinina , Q. E. Barber , et al. 2020. “Fire and Biodiversity in the Anthropocene.” Science 370, no. 6519: eabb0355. 33214246 10.1126/science.abb0355

[gcb70945-bib-0324] Kirchner, J. W. , R. C. Finkel , C. S. Riebe , et al. 2001. “Mountain Erosion Over 10 yr, 10 ky, and 10 My Time Scales.” Geology 29, no. 7: 591–594.

[gcb70945-bib-0144] Klimas, K. B. , L. L. Yocom , B. P. Murphy , et al. 2025. “A Machine Learning Model to Predict Wildfire Burn Severity for Pre‐Fire Risk Assessments, Utah, USA.” Fire Ecology 21, no. 1: 8.

[gcb70945-bib-0145] Klose, K. , S. D. Cooper , and D. M. Bennett . 2015. “Effects of Wildfire on Stream Algal Abundance, Community Structure, and Nutrient Limitation.” Freshwater Science 34, no. 4: 1494–1509.

[gcb70945-bib-0146] Kominoski, J. S. , M. Fernandez , P. Breault , V. Sclater , and B. B. Rothermel . 2022. “Fire Severity and Post‐Fire Hydrology Drive Nutrient Cycling and Plant Community Recovery in Intermittent Wetlands.” Ecosystems 25, no. 2: 265–278.

[gcb70945-bib-0147] Koontz, E. D. , E. A. Steel , and J. D. Olden . 2018. “Stream Thermal Responses to Wildfire in the Pacific Northwest.” Freshwater Science 37, no. 4: 731–746.

[gcb70945-bib-0149] Krasnow, K. D. , A. S. Halford , and S. L. Stephens . 2012. “Aspen Restoration in the Eastern Sierra Nevada: Effectiveness of Prescribed Fire and Conifer Removal.” Fire Ecology 8, no. 3: 104–118.

[gcb70945-bib-0150] Krawchuk, M. A. , M. A. Moritz , M. A. Parisien , J. Van Dorn , and K. Hayhoe . 2009. “Global Pyrogeography: The Current and Future Distribution of Wildfire.” PLoS One 4, no. 4: e5102. 19352494 10.1371/journal.pone.0005102 PMC2662419

[gcb70945-bib-0151] Kreider, M. R. , P. E. Higuera , S. A. Parks , W. L. Rice , N. White , and A. J. Larson . 2024. “Fire Suppression Makes Wildfires More Severe and Accentuates Impacts of Climate Change and Fuel Accumulation.” Nature Communications 15, no. 1: 2412. 10.1038/s41467-024-46702-0 PMC10963776 38528012

[gcb70945-bib-0152] Kreye, J. K. , J. K. Hiers , J. M. Varner , B. Hornsby , S. Drukker , and J. J. O'brien . 2018. “Effects of Solar Heating on the Moisture Dynamics of Forest Floor Litter in Humid Environments: Composition, Structure, and Position Matter.” Canadian Journal of Forest Research 48, no. 11: 1331–1342.

[gcb70945-bib-0154] Kutser, T. 2009. “Passive Optical Remote Sensing of Cyanobacteria and Other Intense Phytoplankton Blooms in Coastal and Inland Waters.” International Journal of Remote Sensing 30, no. 17: 4401–4425.

[gcb70945-bib-0155] Laicher, D. , K. Benkendorff , S. G. Johnston , et al. 2026. “Wildfire Impacts Along the Coastal Aquatic Continuum: Addressing the Estuarine Knowledge Gap.” Marine and Freshwater Research 77, no. 2: MF25043. 10.1071/MF25043.

[gcb70945-bib-0158] Lawrence, A. J. , C. Matuch , J. J. Hancock , A. L. Rypel , and L. A. Eliassen . 2022. “Potential Local Extirpation of an Imperiled Freshwater Mussel Population From Wildfire Runoff.” Western North American Naturalist 82, no. 4: 695–703.

[gcb70945-bib-0159] Leblon, B. , L. Bourgeau‐Chavez , and J. San‐Miguel‐Ayanz . 2012. “Use of Remote Sensing in Wildfire Management.” in Sustainable Development‐Authoritative and Leading Edge Content for Environmental Management, 55–82. Published by IntechOpen.

[gcb70945-bib-0161] Legge, E. O. , A. Koyama , C. W. Fernandez , et al. 2025. “Dearth Under Earth: Understudied Plant‐Soil‐Fire Feedback as Drivers of Forest Mesophication and Oak Regeneration Failures.” Forest Ecology and Management 597: 123147.

[gcb70945-bib-0162] Lentile, L. B. , Z. A. Holden , A. M. Smith , et al. 2006. “Remote Sensing Techniques to Assess Active Fire Characteristics and Post‐Fire Effects.” International Journal of Wildland Fire 15, no. 3: 319–345.

[gcb70945-bib-0163] Lewis, T. L. , M. S. Lindberg , J. A. Schmutz , and M. R. Bertram . 2014. “Multi‐Trophic Resilience of Boreal Lake Ecosystems to Forest Fires.” Ecology 95, no. 5: 1253–1263. 25000757 10.1890/13-1170.1

[gcb70945-bib-0165] Li, X. , K. Song , and G. Liu . 2020. “Wetland Fire Scar Monitoring and Its Response to Changes of the Pantanal Wetland.” Sensors 20, no. 15: 4268. 32751781 10.3390/s20154268 PMC7436325

[gcb70945-bib-0166] Li, X. , G. Wang , Y. Li , D. Han , J. Cong , and C. Gao . 2023. “Aerobic and Anaerobic Burning Alter Trace Metal Availability in Peat Soils: Evidence From Laboratory Experiments.” European Journal of Soil Science 74, no. 3: e13385.

[gcb70945-bib-0325] Lindenmayer, D. B. , E. J. Bowd , C. Taylor , and G. E. Likens . 2022. “The Interactions Among Fire, Logging, and Climate Change Have Sprung a Landscape Trap in Victoria's Montane Ash Forests.” Plant Ecology 223, no. 7: 733–749.

[gcb70945-bib-0167] Lindenmayer, D. B. , P. J. Burton , and J. F. Franklin . 2008. Salvage Logging and Its Ecological Consequences. Island Press 246 pp.

[gcb70945-bib-0168] Link, N. T. , D. L. McLaughlin , N. Bush , and F. C. Wurster . 2024. “Phragmites‐Fire Feedbacks: The Influence of Fire and Disturbance‐Altered Hydrology on the Abundance of *Phragmites australis* .” Biological Invasions 26, no. 1: 135–150.

[gcb70945-bib-0172] Lukenbach, M. C. , K. J. Hokanson , P. A. Moore , et al. 2015. “Hydrological Controls on Deep Burning in a Northern Forested Peatland.” Hydrological Processes 29, no. 18: 4114–4124.

[gcb70945-bib-0503] Luvuno, L. B. , D. C. Kotze , and K. P. Kirkman . 2016. “Long‐Term Landscape Changes in Vegetation Structure: Fire Management in the Wetlands of KwaMbonambi, South Africa.” African Journal of Aquatic Science 41, no. 3: 279–288.

[gcb70945-bib-0173] Lynch, A. J. , S. J. Cooke , A. M. Deines , et al. 2016. “The Social, Economic, and Environmental Importance of Inland Fish and Fisheries.” Environmental Reviews 24, no. 2: 115–121.

[gcb70945-bib-0175] Mackay, K. D. , B. Vincent , M. Southwell , I. Growns , and S. Mika . 2024. “Differential Impacts of Fire and Inundation on a Wetland Plant Community After Wildfire.” Marine and Freshwater Research 75, no. 15: MF24040.

[gcb70945-bib-0326] Malison, R. L. , and C. V. Baxter . 2010. “The Fire Pulse: Wildfire Stimulates Flux of Aquatic Prey to Terrestrial Habitats Driving Increases in Riparian Consumers.” Canadian Journal of Fisheries and Aquatic Sciences 67, no. 3: 570–579.

[gcb70945-bib-0327] Marcotte, A. L. , J. Limpens , J. P. Nunes , et al. 2024. “Enhanced Hydrologic Connectivity and Solute Dynamics Following Wildfire and Drought in a Contaminated Temperate Peatland Catchment.” Water Resources Research 60, no. 7: e2023WR036412.

[gcb70945-bib-0328] Marcotte, A. L. 2025. “From the Ashes: Hydrological and Biogeochemical Responses to Wildfire in Temperate Peatlands.” Doctoral dissertation, Wageningen University and Research.

[gcb70945-bib-0178] Markle, C. E. , and P. Chow‐Fraser . 2018. “Effects of European Common Reed on Blanding's Turtle Spatial Ecology.” Journal of Wildlife Management 82, no. 4: 857–864.

[gcb70945-bib-0179] Markle, C. E. , H. J. Gage , A. M. Tekatch , S. L. Wilkinson , and J. M. Waddington . 2022. “Wetland Successional State Affects Fire Severity in a Boreal Shield Landscape.” Wetlands 42, no. 7: 87.

[gcb70945-bib-0180] Markle, C. E. , P. A. Moore , and J. M. Waddington . 2020. “Primary Drivers of Reptile Overwintering Habitat Suitability: Integrating Wetland Ecohydrology and Spatial Complexity.” Bioscience 70, no. 7: 597–609.

[gcb70945-bib-0181] Markle, C. E. , S. L. Wilkinson , and J. M. Waddington . 2020. “Initial Effects of Wildfire on Freshwater Turtle Nesting Habitat.” Journal of Wildlife Management 84, no. 7: 1373–1383.

[gcb70945-bib-0182] Marques, N. , F. Miranda , L. Gomes , F. Lenti , L. Costa , and M. Bustamante . 2022. “Fire Effects on Riparian Vegetation Recovery and Nutrient Fluxes in Brazilian Cerrado.” Austral Ecology 47, no. 6: 1168–1183.

[gcb70945-bib-0329] Marrs, R. H. , E. L. Marsland , R. Lingard , et al. 2019. “Experimental Evidence for Sustained Carbon Sequestration in Fire‐Managed, Peat Moorlands.” Nature Geoscience 12, no. 2: 108–112.

[gcb70945-bib-0183] Martens, A. M. , U. Silins , H. C. Proctor , et al. 2019. “Long‐Term Impact of Severe Wildfire and Post‐Wildfire Salvage Logging on Macroinvertebrate Assemblage Structure in Alberta's Rocky Mountains.” International Journal of Wildland Fire 28, no. 10: 738–749.

[gcb70945-bib-0185] Mason, T. J. , G. C. Popovic , M. McGillycuddy , and D. A. Keith . 2023. “Effects of Hydrological Change in Fire‐Prone Wetland Vegetation: An Empirical Simulation.” Journal of Ecology 111, no. 5: 1050–1062.

[gcb70945-bib-0186] McCarter, C. P. R. , G. D. Clay , S. L. Wilkinson , et al. 2024. “Peat Fires and Legacy Toxic Metal Release: An Integrative Biogeochemical and Ecohydrological Conceptual Framework.” Earth‐Science Reviews 256: 104867.

[gcb70945-bib-0187] McCarter, C. P. R. , S. L. Wilkinson , P. A. Moore , and J. M. Waddington . 2021. “Ecohydrological Trade‐Offs From Multiple Peatland Disturbances: The Interactive Effects of Drainage, Harvesting, Restoration and Wildfire in a Southern Ontario Bog.” Journal of Hydrology 601: 126793.

[gcb70945-bib-0188] McCullough, I. M. , K. S. Cheruvelil , J. F. Lapierre , et al. 2019. “Do Lakes Feel the Burn? Ecological Consequences of Increasing Exposure of Lakes to Fire in the Continental United States.” Global Change Biology 25, no. 9: 2841–2854. 31301168 10.1111/gcb.14732

[gcb70945-bib-0330] McFarland, J. R. , J. D. Coop , J. A. Balik , K. C. Rodman , S. A. Parks , and C. S. Stevens‐Rumann . 2025. “Extreme Fire Spread Events Burn More Severely and Homogenize Postfire Landscapes in the Southwestern United States.” Global Change Biology 31, no. 2: e70106. 40007450 10.1111/gcb.70106 PMC11862873

[gcb70945-bib-0189] McGuire, L. A. , B. A. Ebel , F. K. Rengers , D. C. Vieira , and P. Nyman . 2024. “Fire Effects on Geomorphic Processes.” Nature Reviews Earth & Environment 5, no. 7: 486–503.

[gcb70945-bib-0190] McIver, J. D. , R. E. J. Boerner , and S. C. Hart . 2008. “The National Fire and Fire Surrogate Study: Ecological Consequences of Alternative Fuel Reduction Methods in Seasonally Dry Forests.” Forest Ecology and Management 255, no. 8–9: 3075–3080.

[gcb70945-bib-0191] McLauchlan, K. K. , P. E. Higuera , J. Miesel , et al. 2020. “Fire as a Fundamental Ecological Process: Research Advances and Frontiers.” Journal of Ecology 108, no. 5: 2047–2069.

[gcb70945-bib-0193] Merschel, A. G. , M. A. Krawchuk , J. D. Johnston , and T. A. Spies . 2024. “Historical Pyrodiversity in Douglas‐Fir Forests of the Southern Cascades of Oregon, USA.” Forest Ecology and Management 572: 122306.

[gcb70945-bib-0194] Minshall, G. W. 2003. “Responses of Stream Benthic Macroinvertebrates to Fire.” Forest Ecology and Management 178, no. 1–2: 155–161.

[gcb70945-bib-0195] Montgomery, D. R. 1999. “Process Domains and the River Continuum.” Journal of the American Water Resources Association 35, no. 2: 397–410.

[gcb70945-bib-0196] Moody, J. A. , and D. A. Martin . 2009. “Synthesis of Sediment Yields After Wildland Fire in Different Rainfall Regimes in the Western United States.” International Journal of Wildland Fire 18, no. 1: 96–115.

[gcb70945-bib-0197] Moore, J. W. , M. E. Ulaski , K. L. Wilson , et al. 2025. “A Safe Operating Space for Salmon Watersheds Under Rapid Climate Change.” Fish and Fisheries 26, no. 6: 1213–1228.

[gcb70945-bib-0198] Moreira, F. , D. Ascoli , H. Safford , et al. 2020. “Wildfire Management in Mediterranean‐Type Regions: Paradigm Change Needed.” Environmental Research Letters 15, no. 1: 011001.

[gcb70945-bib-0199] Morrison, C. , L. F. Grogan , N. Clemann , and C. Lanctôt . 2025. “Impacts of Fire‐Fighting Chemicals on Native Fauna and Ecosystems in Australia: Identification of Key Knowledge Gaps and Research Priorities.” Environmental Management 75, no. 6: 1559–1570. 10.1007/s00267-025-02143-z. 40128459 PMC12084226

[gcb70945-bib-0200] Morvan, D. 2019. “Wildfires Modelling: Short Overview, Challenges and Perspectives.” Journal of the Combustion Society of Japan 61, no. 196: 120–125.

[gcb70945-bib-0201] Murphy, B. P. , J. A. Czuba , and P. Belmont . 2019. “Post‐Wildfire Sediment Cascades: A Modeling Framework Linking Debris Flow Generation and Network‐Scale Sediment Routing.” Earth Surface Processes and Landforms 44, no. 11: 2126–2140.

[gcb70945-bib-0202] Murphy, S. F. , C. N. Alpers , C. W. Anderson , et al. 2023. “A Call for Strategic Water‐Quality Monitoring to Advance Assessment and Prediction of Wildfire Impacts on Water Supplies.” Frontiers in Water 5: 1144225.

[gcb70945-bib-0203] Murphy, S. F. , J. M. Blake , B. A. Ebel , and D. A. Martin . 2025. “Intersection of Wildfire and Legacy Mining Poses Risks to Water Quality.” Environmental Science & Technology 59, no. 1: 35–44. 10.1021/acs.est.4c09489. 39700319 PMC11741108

[gcb70945-bib-0204] Murphy, S. F. , R. B. McCleskey , D. A. Martin , J. M. Holloway , and J. H. Writer . 2020. “Wildfire‐Driven Changes in Hydrology Mobilize Arsenic and Metals From Legacy Mine Waste.” Science of the Total Environment 743: 140635. 32663689 10.1016/j.scitotenv.2020.140635

[gcb70945-bib-0205] National Park Service . 2025. Types of Wildland Fire. U.S. Department of the Interior. https://www.nps.gov/subjects/fire/types‐of‐wildland‐fire.htm.

[gcb70945-bib-0207] Nelson, K. , D. Thompson , C. Hopkinson , R. Petrone , and L. Chasmer . 2021. “Peatland‐Fire Interactions: A Review of Wildland Fire Feedbacks and Interactions in Canadian Boreal Peatlands.” Science of the Total Environment 769: 145212. 33486170 10.1016/j.scitotenv.2021.145212

[gcb70945-bib-0332] Neville, H. , J. Dunham , A. Rosenberger , J. Umek , and B. Nelson . 2009. “Influences of Wildfire, Habitat Size, and Connectivity on Trout in Headwater Streams Revealed by Patterns of Genetic Diversity.” Transactions of the American Fisheries Society 138, no. 6: 1314–1327.

[gcb70945-bib-0208] Ngole‐Jeme, V. M. 2019. “Fire‐Induced Changes in Soil and Implications on Soil Sorption Capacity and Remediation Methods.” Applied Sciences 9, no. 17: 3447.

[gcb70945-bib-0209] Nguyen, T. Q. , H. X. Nguyen , M. Q. Bui , et al. 2025. Towards a Standardized Design Framework for Phytoremediation of Contaminants in Water: Bridging Research and Policy‐Driven Applications.

[gcb70945-bib-0210] Nguyen, V. M. , N. Young , and S. J. Cooke . 2017. “A Roadmap for Knowledge Exchange and Mobilization Research in Conservation and Natural Resource Management.” Conservation Biology 31, no. 4: 789–798. 27767241 10.1111/cobi.12857

[gcb70945-bib-0211] Niemeyer, R. J. , K. D. Bladon , and R. D. Woodsmith . 2020. “Long‐Term Hydrologic Recovery After Wildfire and Post‐Fire Forest Management in the Interior Pacific Northwest.” Hydrological Processes 34: 1182–1197.

[gcb70945-bib-0213] Nocentini, S. , G. Buttoud , O. Ciancio , and P. Corona . 2017. “Managing Forests in a Changing World: The Need for a Systemic Approach. A Review.” Forestry Systems 26: eR01.

[gcb70945-bib-0214] North, T. D. , C. E. Markle , R. Y. Fallas , P. A. Moore , and J. M. Waddington . 2024. “Initial Impacts of Wildfire on Overwintering Conditions for a Species‐At‐Risk Snake.” Global Ecology and Conservation 56: e03258.

[gcb70945-bib-0215] Nunes, B. , V. Silva , I. Campos , et al. 2017. “Off‐Site Impacts of Wildfires on Aquatic Systems—Biomarker Responses of the Mosquitofish *Gambusia holbrooki* .” Science of the Total Environment 581‐582: 305–313. 10.1016/j.scitotenv.2016.12.129 28088544

[gcb70945-bib-0216] Odermatt, D. , A. Gitelson , V. E. Brando , and M. Schaepman . 2012. “Review of Constituent Retrieval in Optically Deep and Complex Waters From Satellite Imagery.” Remote Sensing of Environment 118: 116–126.

[gcb70945-bib-0217] Olson, N. E. , K. L. Boaggio , R. B. Rice , K. M. Foley , and S. D. LeDuc . 2023. “Wildfires in the Western United States Are Mobilizing PM 2.5‐Associated Nutrients and May Be Contributing to Downwind Cyanobacteria Blooms.” Environmental Science: Processes & Impacts 25, no. 6: 1049–1066. 37232758 10.1039/d3em00042g PMC10585592

[gcb70945-bib-0218] Orem, C. A. , and J. D. Pelletier . 2015. “Quantifying the Time Scale of Elevated Geomorphic Response Following Wildfires Using Multi‐Temporal LiDAR Data: An Example From the Las Conchas Fire, Jemez Mountains, New Mexico.” Geomorphology 232: 224–238.

[gcb70945-bib-0219] Pacific Salmon Foundation . 2024. Playbook to Guide Landscape Recovery Strategies & Priorities for Salmon Habitat Following Major Wildfires (Version 1.2, October 2024). Pacific Salmon Foundation. https://psf.ca/wp‐content/uploads/2024/10/21P0581_PSF_Playbook_V1.2_25October2024.pdf.

[gcb70945-bib-0220] Palm, E. C. , M. J. Suitor , K. Joly , et al. 2022. “Increasing Fire Frequency and Severity Will Increase Habitat Loss for a Boreal Forest Indicator Species.” Ecological Applications 32, no. 3: e2549. 10.1002/eap.2549. 35094462 PMC9286541

[gcb70945-bib-0221] Palmer, S. C. , T. Kutser , and P. D. Hunter . 2015. “Remote Sensing of Inland Waters: Challenges, Progress and Future Directions.” Remote Sensing of Environment 157: 1–8.

[gcb70945-bib-0222] Parks Canada . 2025. Indigenous Fire Stewardship. Government of Canada. https://parks.canada.ca/nature/science/conservation/feu‐fire/autochtones‐indigenous.

[gcb70945-bib-0223] Parsons, A. , P. R. Robichaud , S. A. Lewis , C. Napper , and J. T. Clark . 2010. Field Guide for Mapping Post‐Fire Soil Burn Severity. Gen. Tech. Rep. RMRS‐GTR‐243. US Department of Agriculture, Forest Service, Rocky Mountain Research Station, 49 p.

[gcb70945-bib-0224] Paterson, A. M. , D. S. Morimoto , B. F. Cumming , J. P. Smol , and J. M. Szeicz . 2002. “A Paleolimnological Investigation of the Effects of Forest Fire on Lake Water Quality in Northwestern Ontario Over the Past Ca. 150 Years.” Canadian Journal of Botany 80, no. 12: 1329–1336.

[gcb70945-bib-0225] Paul, M. J. , S. D. LeDuc , M. G. Lassiter , L. C. Moorhead , P. D. Noyes , and S. G. Leibowitz . 2022. “Wildfire Induces Changes in Receiving Waters: A Review With Considerations for Water Quality Management.” Water Resources Research 58, no. 9: e2021WR030699. 10.1029/2021wr030699 PMC10034714 36968177

[gcb70945-bib-0226] Pelletier, N. , J. Chételat , O. Blarquez , and J. C. Vermaire . 2020. “Paleolimnological Assessment of Wildfire‐Derived Atmospheric Deposition of Trace Metal (Loid) s and Major Ions to Subarctic Lakes (Northwest Territories, Canada).” Journal of Geophysical Research: Biogeosciences 125, no. 8: e2020JG005720.

[gcb70945-bib-0227] Pennino, M. J. , S. G. Leibowitz , J. E. Compton , M. T. Beyene , and S. D. LeDuc . 2022. “Wildfires Can Increase Regulated Nitrate, Arsenic, and Disinfection Byproduct Violations and Concentrations in Public Drinking Water Supplies.” Science of the Total Environment 804: 149890. 34520927 10.1016/j.scitotenv.2021.149890 PMC10084414

[gcb70945-bib-0228] Peterson, G. D. 2002. “Contagious Disturbance, Ecological Memory, and the Emergence of Landscape Pattern.” Ecosystems 5: 329–338.

[gcb70945-bib-0229] Pilliod, D. S. , R. B. Bury , E. J. Hyde , C. A. Pearl , and P. S. Corn . 2003. “Fire and Amphibians in North America.” Forest Ecology and Management 178, no. 1–2: 163–181.

[gcb70945-bib-0230] Pleizier, N. , G. D. Schwieterman , K. Birnie‐Gauvin , et al. 2025. “Conservation Physiology of Freshwater Fishes: An Illustration of Pressing Questions and Implications for Management. Conservation.” Physiology 13, no. 1: coaf057. 10.1093/conphys/coaf057 PMC12321299 40761532

[gcb70945-bib-0231] Pugh, B. E. , M. Colley , S. J. Dugdale , et al. 2022. “A Possible Role for River Restoration Enhancing Biodiversity Through Interaction With Wildfire.” Global Ecology and Biogeography 31, no. 10: 1990–2004.

[gcb70945-bib-0232] Puglis, H. J. , M. Iacchetta , and C. M. Mackey . 2022. “Toxicity of Wildland Fire‐Fighting Chemicals in Pulsed Exposures to Rainbow Trout and Fathead Minnows.” Environmental Toxicology and Chemistry 41, no. 7: 1711–1720. 10.1002/etc.5347. 35452533

[gcb70945-bib-0234] Reeves, G. H. , P. A. Bisson , B. E. Rieman , and L. E. Benda . 2006. “Postfire Logging in Riparian Areas.” Conservation Biology 20, no. 4: 994–1004. 16922216 10.1111/j.1523-1739.2006.00502.x

[gcb70945-bib-0235] Reilly, M. J. , A. Zuspan , J. S. Halofsky , et al. 2022. “Cascadia Burning: The Historic, but Not Historically Unprecedented, 2020 Wildfires in the Pacific Northwest, USA.” Ecosphere 13: e4070.

[gcb70945-bib-0334] Rengers, F. K. , S. Bower , A. Knapp , et al. 2024. “Evaluating Post‐Wildfire Debris‐Flow Rainfall Thresholds and Volume Models at the 2020 Grizzly Creek Fire in Glenwood Canyon, Colorado, USA.” Natural Hazards and Earth System Sciences 24, no. 6: 2093–2114.

[gcb70945-bib-0237] Rey, D. M. , M. A. Briggs , M. A. Walvoord , and B. A. Ebel . 2023. “Wildfire‐Induced Shifts in Groundwater Discharge to Streams Identified With Paired Air and Stream Water Temperature Analyses.” Journal of Hydrology 619: 129272.

[gcb70945-bib-0238] Rhoades, C. C. , T. S. Fegel , A. E. Rhea , et al. 2025. “Stream Chemistry After Colorado's Largest Wildfire: Solute‐Specific Responses to Ash and Rainstorms.” Ecosystems 28, no. 5: 55.

[gcb70945-bib-0239] Richardson, C. , M. Montalvo , S. Wagner , et al. 2024. “Exploring the Complex Effects of Wildfire on Stream Water Chemistry: Insights From Concentration‐Discharge Relationships.” Water Resources Research 60, no. 2: e2023WR034940.

[gcb70945-bib-0240] Ridgway, P. , B. Lane , H. Canham , B. P. Murphy , P. Belmont , and F. K. Rengers . 2024. “Wildfire, Extreme Precipitation and Debris Flows, Oh My! Channel Response to Compounding Disturbances in a Mountain Stream in the Upper Colorado Basin, USA.” Earth Surface Processes and Landforms 49, no. 12: 3855–3872.

[gcb70945-bib-0241] Rieman, B. E. , P. F. Hessburg , C. Luce , and M. R. Dare . 2010. “Wildfire and Management of Forests and Native Fishes: Conflict or Opportunity for Convergent Solutions?” Bioscience 60, no. 6: 460–468.

[gcb70945-bib-0333] Riera, R. , and J. G. Pausas . 2024. “Fire Ecology in Marine Systems.” Trends in Ecology & Evolution 39, no. 3: 221–224. 38160176 10.1016/j.tree.2023.12.001

[gcb70945-bib-0243] Rinne, J. N. 1996. “Management Briefs: Short‐Term Effects of Wildfire on Fishes and Aquatic Macroinvertebrates in the Southwestern United States.” North American Journal of Fisheries Management 16, no. 3: 653–658.

[gcb70945-bib-0244] River, M. , P. James , M. J. Fix , J. Walter , and T. Renata . 2026. “Effects of Wildfire on Stream Temperatures and Riparian Shade in Managed Timberlands.” Ecohydrology 19, no. 2: e70193.

[gcb70945-bib-0245] Roberts, H. P. , L. L. Willey , M. T. Jones , et al. 2023. “Effects of Landscape Structure and Land Use on Turtle Communities Across the Eastern United States.” Biological Conservation 283: 110088.

[gcb70945-bib-0246] Robichaud, C. D. , and R. C. Rooney . 2022. “Invasive Grass Causes Biotic Homogenization in Wetland Birds in a Lake Erie Coastal Marsh.” Hydrobiologia 849: 3197–3212.

[gcb70945-bib-0247] Roche, M. D. , D. J. Saher , E. K. Buchholtz , et al. 2024. “Ecological Trade‐Offs Associated With Fuel Breaks in Sagebrush Ecosystems.” Fire Ecology 20, no. 1: 107.

[gcb70945-bib-0248] Rogers, B. M. , J. K. Balch , S. J. Goetz , C. E. Lehmann , and M. Turetsky . 2020. “Focus on Changing Fire Regimes: Interactions With Climate, Ecosystems, and Society.” Environmental Research Letters 15, no. 3: 030201.

[gcb70945-bib-0249] Roghair, C. N. , C. A. Dolloff , and M. K. Underwood . 2002. “Response of a Brook Trout Population and Instream Habitat to a Catastrophic Flood and Debris Flow.” Transactions of the American Fisheries Society 131, no. 4: 718–730.

[gcb70945-bib-0335] Romualdi, D. C. , S. L. Wilkinson , and P. M. A. James . 2023. “On the Limited Consensus of Mountain Pine Beetle Impacts on Wildfire.” Landscape Ecology 38, no. 9: 2159–2178. 37521154 10.1007/s10980-023-01720-z PMC10372117

[gcb70945-bib-0250] Roon, D. A. , J. R. Bellmore , J. R. Benjamin , et al. 2025. “Linking Fire, Food Webs, and Fish in Stream Ecosystems.” Ecosystems 28, no. 1: 1. 39759976 10.1007/s10021-024-00955-4 PMC11698785

[gcb70945-bib-0251] Rosa, J. M. , J. T. Anderson , and S. A. Welsh . 2023. “Wildfire Impacts on Wetland Ecosystems: Hydrological, Vegetation, and Faunal Responses in Freshwater Landscapes.” Wetlands Ecology and Management 31: 215–230.

[gcb70945-bib-0252] Rosenberger, A. E. , J. B. Dunham , J. R. Neuswanger , and S. F. Railsback . 2015. “Legacy Effects of Wildfire on Stream Thermal Regimes and Rainbow Trout Ecology: An Integrated Analysis of Observation and Individual‐Based Models.” Freshwater Science 34, no. 4: 1571–1584.

[gcb70945-bib-0253] Rugenski, A. T. , and G. W. Minshall . 2014. “Climate‐Moderated Responses to Wildfire by Macroinvertebrates and Basal Food Resources in Montane Wilderness Streams.” Ecosphere 5, no. 3: 1–24.

[gcb70945-bib-0254] Russell, K. R. , D. H. Van Lear , and D. C. Guynn Jr. 1999. “Prescribed Fire Effects on Herpetofauna: Review and Management Implications.” Wildlife Society Bulletin 27, no. 2: 374–384.

[gcb70945-bib-0255] Rust, A. J. , T. S. Hogue , S. Saxe , and J. McCray . 2018. “Post‐Fire Water‐Quality Response in the Western United States.” International Journal of Wildland Fire 27, no. 3: 203–216.

[gcb70945-bib-0256] Rust, A. J. , J. Randell , A. S. Todd , and T. S. Hogue . 2019. “Wildfire Impacts on Water Quality, Macroinvertebrate, and Trout Population in the Upper Rio Grande.” Forest Ecology and Management 453: 117636.

[gcb70945-bib-0258] Saab, V. A. , and H. D. Powell . 2005. “Fire and Avian Ecology in North America: Process Influencing Pattern.” Fire and Avian Ecology in North America. Studies in Avian Biology 30: 1–13.

[gcb70945-bib-0259] Sánchez‐García, C. , C. Santín , J. Neris , et al. 2023. “Chemical Characteristics of Wildfire Ash Across the Globe and Their Environmental and Socio‐Economic Implications.” Environment International 178: 108065. 10.1016/j.envint.2023.108065. 37562341

[gcb70945-bib-0260] Sanderfoot, O. V. , S. B. Bassing , J. L. Brusa , et al. 2021. “A Review of the Effects of Wildfire Smoke on the Health and Behavior of Wildlife.” Environmental Research Letters 16, no. 12: 123003.

[gcb70945-bib-0261] Sanders, A. M. , A. A. Coble , A. G. Swartz , M. River , P. James , and D. R. Warren . 2022. “Heat and Smoke From Wildfires Influence Water Temperature and Dissolved Oxygen Levels in Headwater Streams.” Freshwater Science 41, no. 4: 665–679. 10.1086/722632.

[gcb70945-bib-0262] Schäfer, R. B. , L. Hearn , B. J. Kefford , J. F. Mueller , and D. Nugegoda . 2010. “Using Silicone Passive Samplers to Detect Polycyclic Aromatic Hydrocarbons From Wildfires in Streams and Potential Acute Effects for Invertebrate Communities.” Water Research 44, no. 15: 4590–4600. 10.1016/j.watres.2010.05.044. 20554305

[gcb70945-bib-0336] Scheffer, M. 2001. “Stochastic Events can Trigger Large State Shifts in Ecosystems With Reduced Resilience.” 40 Years Theory and Model at Wageningen UR, 47.

[gcb70945-bib-0263] Scheffer, M. , and E. Jeppesen . 2007. “Regime Shifts in Shallow Lakes.” Ecosystems 10, no. 1: 1–3.

[gcb70945-bib-0501] Scheffer, M. 2009. Critical Transitions in Nature and Society. Vol. 16. Princeton University Press.

[gcb70945-bib-0265] Schindler, D. W. 2001. “The Cumulative Effects of Climate Warming and Other Human Stresses on Canadian Freshwaters in the New Millennium.” Canadian Journal of Fisheries and Aquatic Sciences 58, no. 1: 18–29.

[gcb70945-bib-0264] Schindler, D. W. , and J. P. Smol . 2006. “Cumulative Effects of Climate Warming and Other Human Activities on Freshwaters of Arctic and Subarctic North America.” Ambio: A Journal of the Human Environment 35, no. 4: 160–168. 10.1579/0044-7447(2006)35[160:ceocwa]2.0.co;2 16944640

[gcb70945-bib-0266] Schultz, C. A. , L. F. Miller , S. M. Greiner , and C. Kooistra . 2021. “A Qualitative Study on the US Forest Service's Risk Management Assistance Efforts to Improve Wildfire Decision‐Making.” Forests 12, no. 3: 344.

[gcb70945-bib-0267] Schultz, C. A. , M. P. Thompson , and S. M. McCaffrey . 2019. “Forest Service Fire Management and the Elusiveness of Change.” Fire Ecology 15, no. 1: 13.

[gcb70945-bib-0268] Scordo, F. , S. Chandra , E. Suenaga , et al. 2021. “Smoke From Regional Wildfires Alters Lake Ecology.” Scientific Reports 11, no. 1: 10922. 34035357 10.1038/s41598-021-89926-6 PMC8149697

[gcb70945-bib-0269] Sedell, E. R. , R. E. Gresswell , and T. E. McMahon . 2015. “Predicting Spatial Distribution of Postfire Debris Flows and Potential Consequences for Native Trout in Headwater Streams.” Freshwater Science 34, no. 4: 1558–1570.

[gcb70945-bib-0271] Silins, U. , K. D. Bladon , E. N. Kelly , et al. 2014. “Five‐Year Legacy of Wildfire and Salvage Logging Impacts on Nutrient Runoff and Aquatic Plant, Invertebrate, and Fish Productivity.” Ecohydrology 7, no. 6: 1508–1523.

[gcb70945-bib-0272] Singh, H. , L. M. Ang , T. Lewis , et al. 2024. “Trending and Emerging Prospects of Physics‐Based and ML‐Based Wildfire Spread Models: A Comprehensive Review.” Journal of Forestry Research 35, no. 1: 135.

[gcb70945-bib-0273] Smith, H. G. , G. J. Sheridan , P. N. J. Lane , P. Nyman , and S. Haydon . 2011. “Wildfire Effects on Water Quality in Forest Catchments: A Review With Implications for Water Supply.” Journal of Hydrology 396, no. 1: 170–192.

[gcb70945-bib-0274] Smits, A. P. , F. Scordo , M. Tang , et al. 2024. “Wildfire Smoke Reduces Lake Ecosystem Metabolic Rates Unequally Across a Trophic Gradient.” Communications Earth & Environment 5, no. 1: 265.

[gcb70945-bib-0275] Spinti, R. A. , L. E. Condon , and J. Zhang . 2023. “The Evolution of Dam Induced River Fragmentation in the United States.” Nature Communications 14, no. 1: 3820. 10.1038/s41467-023-39194-x PMC10307825 37380647

[gcb70945-bib-0337] Staley, D. M. , J. W. Kean , S. H. Cannon , K. M. Schmidt , and J. L. Laber . 2013. “Objective Definition of Rainfall Intensity–Duration Thresholds for the Initiation of Post‐Fire Debris Flows in Southern California.” Landslides 10, no. 5: 547–562.

[gcb70945-bib-0338] Staley, D. M. , J. A. Negri , J. W. Kean , J. L. Laber , A. C. Tillery , and A. M. Youberg . 2017. “Prediction of Spatially Explicit Rainfall Intensity–Duration Thresholds for Post‐Fire Debris‐Flow Generation in the Western United States.” Geomorphology 278: 149–162.

[gcb70945-bib-0276] Stankova, N. 2023. “Post‐Fire Recovery Monitoring Using Remote Sensing: A Review.” Aerospace Research in Bulgaria 35: 192–200.

[gcb70945-bib-0277] Strayer, D. L. , and D. Dudgeon . 2010. “Freshwater Biodiversity Conservation: Recent Progress and Future Challenges.” Journal of the North American Benthological Society 29, no. 1: 344–358.

[gcb70945-bib-0278] Struecker, B. P. , J. R. Milanovich , M. McIntosh , M. B. Berg , and M. E. Hopton . 2021. “Selective Predation by Pond‐Breeding Salamanders in Ephemeral Wetlands of Ohio and Illinois.” Journal of Herpetology 55, no. 3: 222–228. 10.1670/19-126 PMC8687147 34937953

[gcb70945-bib-0279] Sullivan, A. L. 2009. “Wildland Surface Fire Spread Modelling, 1990–2007. 2: Empirical and Quasi‐Empirical Models.” International Journal of Wildland Fire 18, no. 4: 369–386.

[gcb70945-bib-0280] Sutton, O. F. , A. K. Furukawa , P. A. Moore , P. J. Morris , and J. M. Waddington . 2025. “Shallow Peatlands as Sentinels of Climate Change.” Environmental Research Letters 20, no. 6: 061001.

[gcb70945-bib-0281] Swartz, A. , and D. Warren . 2022. “Wildfire in Western Oregon Increases Stream Temperatures, Benthic Biofilms, and Juvenile Coastal Cutthroat Trout Size and Densities With Mixed Effects on Adult Trout and Coastal Giant Salamanders.” Canadian Journal of Fisheries and Aquatic Sciences 80, no. 3: 503–516.

[gcb70945-bib-0282] Swartz, A. G. , A. A. Coble , B. E. Penaluna , R. L. Flitcroft , J. L. Ebersole , and M. A. Krawchuk . 2025. “Following Megafires Fishes Thrive and Amphibians Persist Even in Severely Burned Watersheds.” Communications Earth & Environment 6, no. 1: 945. 42089106 10.1038/s43247-025-02893-y PMC13138112

[gcb70945-bib-0283] Talucci, A. C. , L. M. Matosziuk , J. A. Hatten , and M. A. Krawchuk . 2020. “An Added Boost in Pyrogenic Carbon When Wildfire Burns Forest With High Pre‐Fire Mortality.” Fire Ecology 16: 21.

[gcb70945-bib-0339] Tekatch, A. M. , C. E. Markle , S. L. Wilkinson , C. P. McCarter , P. A. Moore , and J. M. Waddington . 2025. “Ecohydrological Drivers of Boreal Shield Peatland Fire Refugia.” Ecohydrology 18, no. 5: e70075.

[gcb70945-bib-0284] Tepley, A. J. , F. J. Swanson , and T. A. Spies . 2013. “Fire‐Mediated Pathways of Stand Development in Douglas‐Fir/Western Hemlock Forests of the Pacific Northwest, USA.” Ecology 94: 1729–1743. 24015517 10.1890/12-1506.1

[gcb70945-bib-0285] Thompson, D. K. , D. Schroeder , S. L. Wilkinson , et al. 2020. “Recent Crown Thinning in a Boreal Black Spruce Forest Does Not Reduce Spread Rate nor Total Fuel Consumption: Results From an Experimental Crown Fire in Alberta, Canada.” Fire 3, no. 3: 28.

[gcb70945-bib-0286] Tickner, D. , J. J. Opperman , R. Abell , et al. 2020. “Bending the Curve of Global Freshwater Biodiversity Loss: An Emergency Recovery Plan.” Bioscience 70, no. 4: 330–342. 32284631 10.1093/biosci/biaa002 PMC7138689

[gcb70945-bib-0287] Tonkin, J. D. , T. Siqueira , J. Merder , et al. 2026. “Extreme Events and River Biodiversity Under Climate Change.” Nature Reviews Biodiversity 2: 150–169. 10.1038/s44358-026-00131-7.

[gcb70945-bib-0288] Turner, M. G. 1989. “Landscape Ecology: The Effect of Pattern on Process.” Annual Review of Ecology and Systematics 20: 171–197.

[gcb70945-bib-0290] Vannote, R. L. , G. W. Minshall , K. W. Cummins , J. R. Sedell , and C. E. Cushing . 1980. “The River Continuum Concept.” Canadian Journal of Fisheries and Aquatic Sciences 37: 130–137.

[gcb70945-bib-0340] Verkaik, I. , M. Vila‐Escale , M. Rieradevall , et al. 2015. “Stream Macroinvertebrate Community Responses to Fire: Are They the Same in Different Fire‐Prone Biogeographic Regions?” Freshwater Science 34, no. 4: 1527–1541.

[gcb70945-bib-0294] Vieira, N. K. , W. H. Clements , L. S. Guevara , and B. F. Jacobs . 2004. “Resistance and Resilience of Stream Insect Communities to Repeated Hydrologic Disturbances After a Wildfire.” Freshwater Biology 49, no. 10: 1243–1259.

[gcb70945-bib-0295] Waddington, J. M. , P. J. Morris , N. Kettridge , G. Granath , D. K. Thompson , and P. A. Moore . 2015. “Hydrological Feedbacks in Northern Peatlands.” Ecohydrology 8, no. 1: 113–127.

[gcb70945-bib-0341] Walker, X. , K. Okano , L. Berner , et al. 2023. “Shifts in Ecological Legacies Support Hysteresis of Stand Type Conversions in Boreal Forests.” Ecosystems 26: 1796–1805.

[gcb70945-bib-0342] Wall, S. , M. Snyder , R. Brown , et al. 2025. “Effects of Wildfire on Streambed Sediment in the Cascades and Klamath Regions of the Pacific Northwest.” Research Square. 10.21203/rs.3.rs-7359437/v1.

[gcb70945-bib-0299] Wan, S. , D. Hui , and Y. Luo . 2001. “Fire Effects on Nitrogen Pools and Dynamics in Terrestrial Ecosystems: A Meta‐Analysis.” Ecological Applications 11, no. 5: 1349–1365.

[gcb70945-bib-0300] Waples, R. S. , G. R. Pess , and T. Beechie . 2008. “Evolutionary History of Pacific Salmon in Dynamic Environments.” Evolutionary Applications 1, no. 2: 189–206. 25567626 10.1111/j.1752-4571.2008.00023.x PMC3352440

[gcb70945-bib-0301] Warren, D. R. , D. A. Roon , A. G. Swartz , and K. D. Bladon . 2022. “Loss of Riparian Forests From Wildfire Led to Increased Stream Temperatures in Summer, Yet Salmonid Fish Persisted.” Ecosphere 13, no. 9: e4233.

[gcb70945-bib-0302] Wasserman, T. N. , and S. E. Mueller . 2023. “Climate Influences on Future Fire Severity: A Synthesis of Climate‐Fire Interactions and Impacts on Fire Regimes, High‐Severity Fire, and Forests in the Western United States.” Fire Ecology 19, no. 1: 43.

[gcb70945-bib-0303] Waters, M. N. , J. M. Smoak , and R. S. Vachula . 2023. “Linking Prescribed Fire, Nutrient Deposition and Cyanobacteria Dominance Through Pyroeutrophication in a Subtropical Lake Ecosystem From the Mid Holocene to Present.” Anthropocene 44: 100420.

[gcb70945-bib-0305] Whitney, J. E. , K. B. Gido , T. J. Pilger , D. L. Propst , and T. F. Turner . 2015. “Consecutive Wildfires Affect Stream Biota in Cold‐ and Warmwater Dryland River Networks.” Freshwater Science 34, no. 4: 1510–1526.

[gcb70945-bib-0343] Wilkinson, S. L. , A. K. Furukawa , B. M. Wotton , and J. M. Waddington . 2021. “Mapping Smouldering Fire Potential in Boreal Peatlands and Assessing Interactions With the Wildland–Human Interface in Alberta, Canada.” International Journal of Wildland Fire 30, no. 7: 552–563.

[gcb70945-bib-0306] Wilkinson, S. L. , P. A. Moore , M. D. Flannigan , B. M. Wotton , and J. M. Waddington . 2018. “Did Enhanced Afforestation Cause High Severity Peat Burn in the Fort McMurray Horse River Wildfire?” Environmental Research Letters 13, no. 1: 014018.

[gcb70945-bib-0307] Wilkinson, S. L. , P. A. Moore , D. K. Thompson , et al. 2018. “The Effects of Black Spruce Fuel Management on Surface Fuel Condition and Peat Burn Severity in an Experimental Fire.” Canadian Journal of Forest Research 48, no. 12: 1433–1440.

[gcb70945-bib-0308] Wilkinson, S. L. , A. M. Tekatch , C. E. Markle , P. A. Moore , and J. M. Waddington . 2020. “Shallow Peat Is Most Vulnerable to High Peat Burn Severity During Wildfire.” Environmental Research Letters 15, no. 10: 104032.

[gcb70945-bib-0309] Wilkinson, S. L. , G. J. Verkaik , P. A. Moore , and J. M. Waddington . 2020. “Threshold Peat Burn Severity Breaks Evaporation‐Limiting Feedback.” Ecohydrology 13, no. 1: e2168.

[gcb70945-bib-0310] Wong, C. , L. Ignace , G. Johnson , K. Hicks , and H. Swanson . 2025. “Reflecting on the “10 Calls to Action to Natural Scientists” 5 Years Later: How Do We Keep Moving Forward on Reconciliation?” Facets 10: 1–17.

[gcb70945-bib-0311] Wu, Y. , X. Xu , C. P. R. McCarter , et al. 2022. “Assessing Leached TOC, Nutrients and Phenols From Peatland Soils After Lab‐Simulated Wildfires: Implications to Source Water Protection.” Science of the Total Environment 822: 153579. 35114220 10.1016/j.scitotenv.2022.153579

[gcb70945-bib-0312] Wu, Y. , N. Zhang , G. Slater , J. M. Waddington , and C.‐F. de Lannoy . 2020. “Hydrophobicity of Peat Soils: Characterization of Organic Compound Changes Associated With Heat‐Induced Water Repellency.” Science of the Total Environment 714: 136444. 31986381 10.1016/j.scitotenv.2019.136444

[gcb70945-bib-0313] Zald, H. S. J. , and C. J. Dunn . 2018. “Severe Fire Weather and Intensive Forest Management Increase Fire Severity in a Multi‐Ownership Landscape.” Ecological Applications 28, no. 14: 1068–1080. 29698575 10.1002/eap.1710

[gcb70945-bib-0314] Zhao, C. , J. Xu , D. Shang , et al. 2021. “Application of Constructed Wetlands in the PAH Remediation of Surface Water: A Review.” Science of the Total Environment 780: 146605. 34030309 10.1016/j.scitotenv.2021.146605

